# Catalytic C–H aerobic and oxidant-induced oxidation of alkylbenzenes (including toluene derivatives) over VO^2+^ immobilized on core–shell Fe_3_O_4_@SiO_2_ at room temperature in water†

**DOI:** 10.1039/d0ra03483e

**Published:** 2020-06-22

**Authors:** Pegah Mohammadpour, Elham Safaei

**Affiliations:** Department of Chemistry, College of Sciences, Shiraz University Shiraz 7194684795 Iran e.safae@shirazu.ac.ir

## Abstract

Direct C–H bond oxidation of organic materials, and producing the necessary oxygenated compounds under mild conditions, has attracted increasing interest. The selective oxidation of various alkylbenzenes was carried out by means of a new catalyst containing VO^2+^ species supported on silica-coated Fe_3_O_4_ nanoparticles using *t*-butyl hydroperoxide as an oxidant at room temperature in H_2_O or solvent-free media. The chemical and structural characterization of the catalyst using several methods such as FTIR spectroscopy, XRD, FETEM, FESEM, SAED, EDX and XPS showed that VO^2+^ is covalently bonded to the silica surface. High selectivity and excellent conversion of various toluene derivatives, with less reactive aliphatic (sp^3^) C–H bonds, to related benzoic acids were quite noticeable. The aerobic oxygenation reaction of these alkylbenzenes was studied under the same conditions. All the results accompanied by sustainability of the inexpensive and simple magnetically separable heterogeneous catalyst proved the important criteria for commercial applications.

## Abstract 2

A highly efficient, recoverable, sustainable, economic and eco-friendly catalyst containing VO^2+^ species supported on SiO_2_@Fe_3_O_4_ nanoparticles for selective oxidation of alkylbenzenes using TBHP or O_2_ at room temperature in H_2_O or solvent-free media.

## Graphical abstract

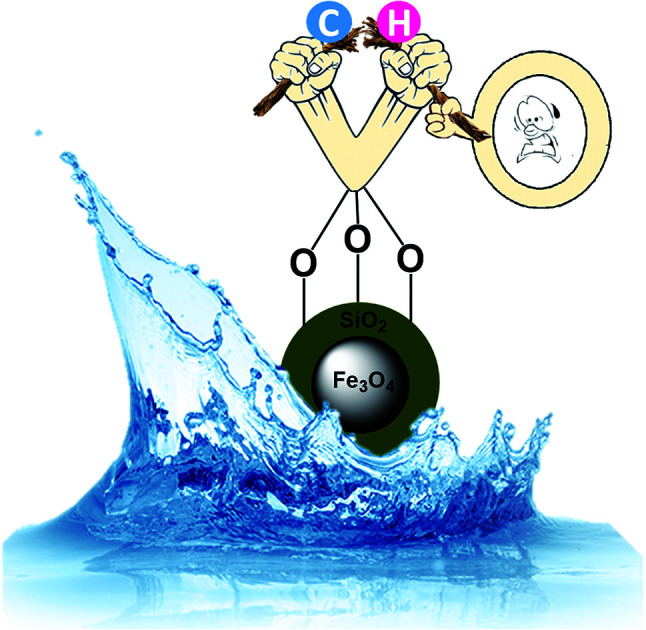

## Introduction

One of the most attractive and valuable fields in chemistry is the direct functionalization of C–H bonds.^1^ Generally, this type of bond is very strong and relatively unreactive, and can be found in organic materials and, in special cases, in natural hydrocarbons and petroleum contaminants. Hence, the activation of C–H bonds in these kinds of materials and consequent transformation of these worthless materials into useful and valuable compounds is a significant economical and green step in synthetic chemistry.^2^ Actually, if C–H bonds are activated selectively, the multistep syntheses of complex molecules can be transformed into facile one-step syntheses. In addition, waste reduction can be supposed from this straightforward and ecofriendly synthetic tool, which makes it a long-standing goal to find new organic scaffolds.^3^ It is worth mentioning that, because of high C–H bond dissociation energy, especially in terminal methyl groups, functionalization of C–H bonds is usually carried out in harsh conditions such as high temperatures, acidic or basic environment, and using strong oxidants or reductants.^4^ Therefore, designing a potential catalyst to achieve the goals of good C–H bond activation efficiency, low cost and low energy consumption is a challenging goal for scientific and industrial communities. Among various hydrocarbons, the benzene family and some of its structural relatives including toluene, ethylbenzene, and xylenes are members of the BTEX group of pollutants which are simply released into the environment by the petroleum and chemical industries and combustion processes.^5^ The selective aerobic oxidation of these compounds to their corresponding alcohol, aldehyde or acid would be a very desirable but challenging research project.^6^ Metal oxides often catalyze these types of reactions at high temperatures in which regulating the reactivity of oxygen is difficult.^7^ For example, bimetallic Pd/Au catalysts were developed to promote the air oxidation of toluene to provide suitable performance at a temperature of 160 °C.^8^ Solvent-free aerobic oxidation of toluene was investigated using anion modified mesoporous mixed oxides at a high temperature of 120 °C and 6 h with low toluene to benzaldehyde conversion value of 15.5%.^9^ Recently, selective C–H oxidation of toluene to benzaldehyde using manganese tungstate (MnWO_4_) nanobars in the presence of H_2_O_2_ as oxidant at 80 °C has been reported.^10^ It has been shown that the most effective transition metals such as palladium,^11^ platinum,^12^ rhenium,^13^ iridium,^14^ ruthenium^15^ and gold^16^ facilitate these efficient transformations through C–H activation pathways. However, the relatively high price, low natural abundance and partly strong toxicity have limited their application. Generally, in chemical reactions, heterogeneous catalysts have advantages over homogeneous catalysts due to their recyclability, easy separation from the reaction mixture and cost effectiveness which make them environmentally benign catalysts. One of the most important classes of heterogeneous catalysts is easily recoverable and reusable magnetic nanoparticles. The most popular magnetic nanoparticles are silica-coated iron oxide nanoparticles because of their high abundance, good physical and thermal stability against degradation, having dielectric property and hydrophobicity, safety and cheapness. Additionally, they are biocompatible and allow easy surface modification due to the presence of abundant silanol groups on their surface.^17^ Supported vanadium oxide catalysts constitute a very important class of heterogeneous catalysts that exhibit a notable ability to catalyze various reactions such as oxidation of sulfides,^18^ oxidative dehydrogenation,^19^ reduction of toxic NO_*x*_^20^ and epoxidation of olefins.^21^ On the other hand, some types of C–H functionalization have been reported using these catalysts as uneconomical reactions which often proceed in difficult situations with relatively low conversion and selectivity with high energy consumption.^22^ According to mentioned considerations, this research is concerned with a newly synthesized vanadyl species (VO^2+^) supported on silica-coated magnetic nanoparticles and its catalytic property evaluation toward C–H activation of alkylbenzenes (especially toluene derivatives).

## Results and discussion

### Catalyst overview

**Scheme 1:**
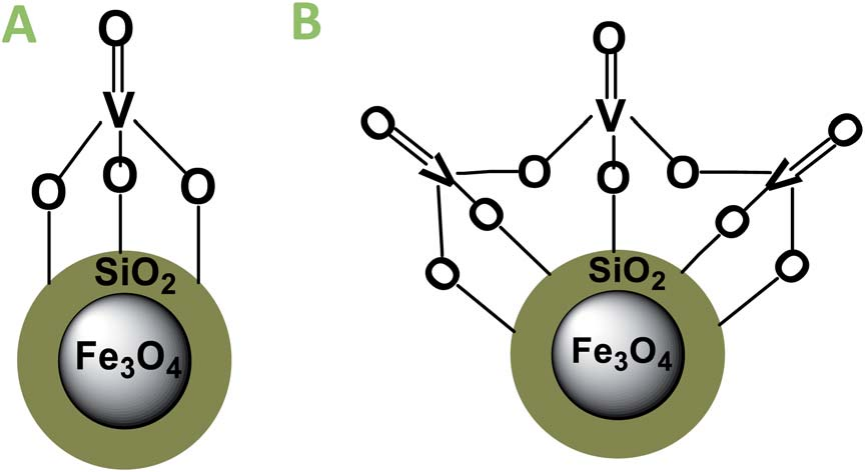
Molecular structures of supported VO_4_^2+^ species. (A) Monomer struture and (B) oligomer structure.

Recently, the attention of research in green chemistry has focused on sustainable and recyclable catalysts which possess high activity and selectivity, cost effectiveness, and are easily available and structurally simple. Concerning the concept of green catalysts, silica-coated Fe_3_O_4_ magnetic nanoparticles (SiO_2_@Fe_3_O_4_) were synthesized by co-precipitation method and coated with silica. The presence of –OH groups on the silica surface and the oxophilic character of vanadium facilitated the immobilization of VO^2+^ species and produced VO^2+^ supported on silica-coated Fe_3_O_4_ (VO^2+^@SiO_2_@Fe_3_O_4_). It has been reported that vanadium species can immobilize on silica surfaces in different forms.^23^ The monomeric form of VO_4_^2+^ species has a terminal V=O and three V–O legs connected to the silica surface (Scheme 1A). Upon increasing the vanadium loading on silica surface, the vanadium species aggregate to oligomer species connected by V–O–V bridges (Scheme 1B) but the isolated monomeric species are supposed to be the most active species during the catalytic process owing to the higher accessibility of the vanadium site compared to other polymeric types.

### Catalyst characterization

**Fig. 1:**
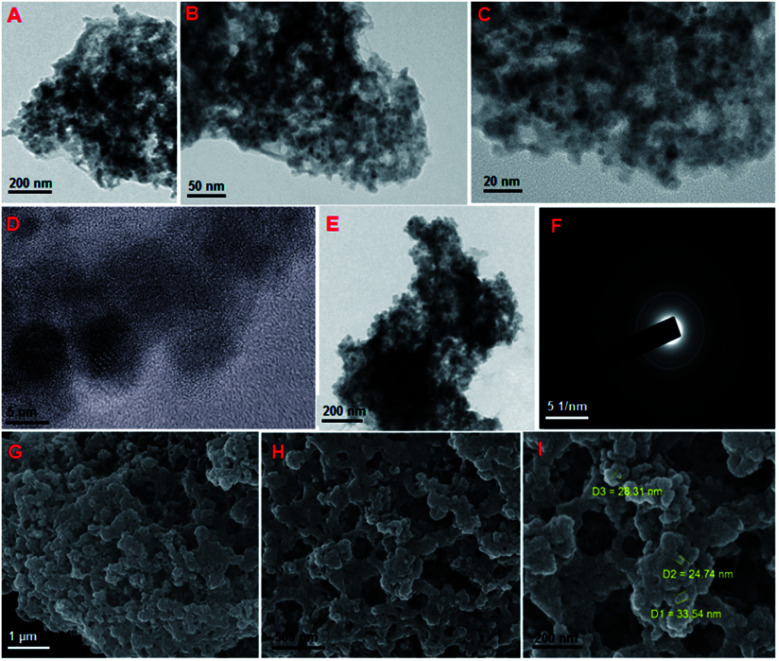
FETEM images of VO^2+^@SiO_2_@Fe_3_O_4_: (A) 200 nm, (B) 50 nm, (C) 20 nm, (D) 5 nm, (E) after recycling. (F) SAED image and FESEM images of the catalyst: (G) 1 μm, (H) 500 nm and (I) 200 nm.

The FT-IR spectra of (A) SiO_2_@Fe_3_O_4_ and (B) VO^2+^@SiO_2_@Fe_3_O_4_ magnetic nanoparticles are shown in Fig. S1.† The bands around 466, 570–616 and 800–1300 cm^−1^ were assigned to the stretching vibrations of symmetric Si–O–Si, Fe–O and symmetric and asymmetric Si–O–Si bonds, respectively. Broad absorption bands of OH groups appeared at 3260–3572 cm^−1^ and the band centered around 1622 cm^−1^ is assigned to the bending vibrations of water. The stabilization of vanadyl species on magnetic nanoparticles was confirmed by the existence of a weak shoulder of V=O stretching at 962 cm^−1^ as reported in the literature.^24^ The crystalline structure of VO^2+^@SiO_2_@Fe_3_O_4_ was explored by using powder XRD (Fig. S2†). All the diffraction peaks corresponded to (220), (311), (400), (422), (511) and (411) pattern, which indicated the cubic spinel crystalline structure of Fe_3_O_4_ according to standard data (JCPDS 19-0629).^25^ This could confirm the stability of the magnetic nanoparticles after coating with silica and immobilization of VO^2+^ species. To characterize the morphology of the catalyst, field-emission transmission electron microscopy (FETEM) and field-emission scanning electron microscopy (FESEM) were applied at different scales (Fig. 1). The uniform morphology of the catalyst is visible in both FETEM and FESEM images. Possibly van der Waals forces between particles lead to an aggregation process. Fig. 1F shows the selected area electron diffraction (SAED) pattern which confirms the cubic structure of magnetic nanoparticles. The elemental composition of VO^2+^@SiO_2_@Fe_3_O_4_ was characterized by energy-dispersive X-ray spectroscopy (EDX) (Fig. S3†) which determined the components of the catalyst. Also, the amount of vanadium incorporated on the silica-coated Fe_3_O_4_ nanoparticles was estimated to be 10.3 mmol g^−1^ by inductively coupled plasma optical emission spectroscopy (ICP-OES) analysis which confirmed the EDX results. Of note, due to the noticeable oxophilicity properties of vanadium especially in higher oxidation states,^26^ the notable percentage of vanadium loading is expected. On the other hand, wasting of VOCl_3_ has been prevented remarkably by the great affinity of vanadyl ions for binding on silica surfaces.

**Fig. 2:**
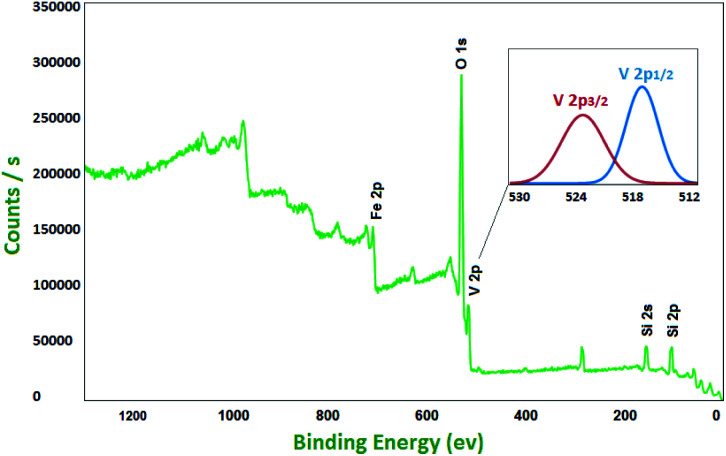
The XPS spectrum of VO^2+^@SiO_2_@Fe_3_O_4_.

Magnetization curve and the hysteresis loops of VO^2+^@SiO_2_@Fe_3_O_4_ show the superparamagnetic behaviour of the catalyst. As shown in Fig. S4,† the magnetic saturation value of magnetic nanoparticles is 18 emu g^−1^. It can be seen that the nanomaterials could be efficiently separated from solution by placing an external magnet near the vessel. For further characterization of the catalyst and investigation of vanadium valence state, X-ray photoelectron spectroscopy (XPS) was used (Fig. 2). A V 2p split was created by spin orbit coupling.^27^ The binding energy levels of 517.7 and 524.8 eV are attributed to V 2p_3/2_ and V 2p_1/2_, respectively, which match well with the literature value for V^5+^.^28^ In the XPS survey of catalyst, peaks related to Fe 2p, O 1s, Si 2s and Si 2p verify the presence of these elements in the catalyst.

### Catalyst activity

#### A. Oxidant induced oxidation of alkylbenzenes

It is noteworthy that toluene remains the most inexpensive and abundant primary source of the aromatic ring in petroleum industries and its transformation to benzoic acid is an important precursor for many other industries. Due to the high reactivity of VO^2+^ species, we carried out economically and in an eco-friendly manner C–H oxidation reactions under different mild conditions to produce oxygenated compounds. Performing these difficult reactions at room temperature and in the absence of organic solvents is of importance and desirable. Selection of a green oxygen source was challenging due to the degradation of H_2_O_2_ by the catalyst, the most common and applicable green oxidant in the vicinity of the catalyst which impelled us to use *t*-butyl hydroperoxide (TBHP) as the main source of oxygen. Toluene oxidation was performed in the presence of various ratios of TBHP, with different solvent systems and at room temperature. In the absence of catalyst, the reaction was stopped with only 5% conversion of toluene (Table 1, entry 1). Increasing amounts of catalyst (mol%) and TBHP equiv. did not improve the conversion and selectivity markedly (Table 1, entries 2–5). Also, performing the reaction under oxygen atmosphere did not allow it to proceed further. Among different solvent systems and conditions, the best result was obtained in water (0.5 mL), with 2 equiv. TBHP and 40 mg catalyst (52 mol%). It seems that oxygen blocked the path of the reaction because the best percentage conversion and selectivity was gained under argon atmosphere. It is worth noting that the applied high mol% of catalyst in the absence of ligand assistance is reasonable.

**Table 1:** Optimization of toluene oxidation reaction

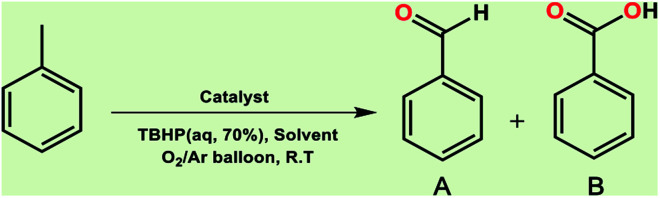							
Entry	Catalyst [mg, mol%]	Solvent	O_2_/Ar	TBHP [equiv.]	*T* [h]	Conv. [%]	Sel. [%] to B
1	—	Solvent free	O_2_	2	9	5	97
2	30, 39	Solvent free	O_2_	2	9	64	51
3	40, 52	Solvent free	O_2_	2	9	81	50
4	60, 78	Solvent free	O_2_	2	9	79	55
5	40, 52	Solvent free	O_2_	4	9	85	42
6	40, 52	Solvent free	Ar	2	9	81	50
7	40, 52	CH_3_CN	Ar	2	9	90	60
8	40, 52	H_2_O	Ar	2	9	99	99

Optimization of conditions for other alkylbenzenes was performed in different situations. Ethylbenzene was chosen as a typical reactant for this reaction (Table 2). In the absence of catalyst, no progress was seen in ethylbenzene oxidation (Table 2, entry 1). Unlike toluene derivatives, a higher level of conversion and selectivity for solvent-free oxidation reactions was seen (Table 2, entries 2–6) and the green solvent systems, especially water, did not help to improve the reaction process (Table 2, entries 8–10). In addition, improvement in the conversion to oxygenated products was observed in the presence of oxygen balloon and not argon atmosphere (Table 2, entry 7), which suggests a different mechanism from that of toluene family. The efficiency of the catalyst in conversion of toluene was examined in the presence of various toluene derivatives including different electron-donating and electron-withdrawing substituents under optimized conditions (Table 3, entries 1–13).

**Table 2:** Optimization of ethylbenzene oxidation reaction

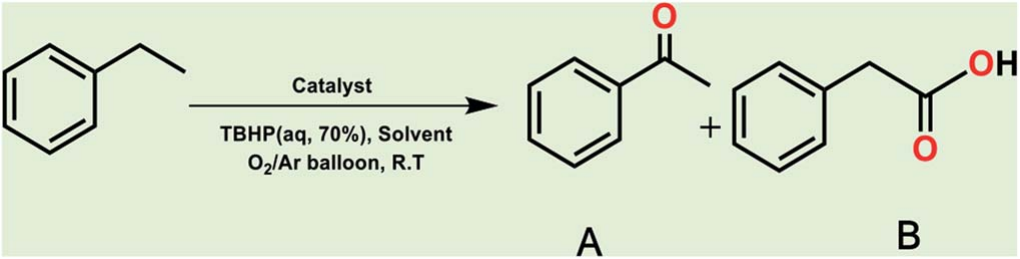							
Entry	Catalyst [mg, mol%]	Solvent	O_2_/Ar	TBHP [equiv.]	*T* [h]	Conv. [%]	Sel. [%] to A
1	—	Solvent free	O_2_	2	8	4	97
2	30, 39	Solvent free	O_2_	2	8	53	93
3	40, 52	Solvent free	O_2_	2	8	>99	99
4	50, 65	Solvent free	O_2_	2	8	92	87
5	40, 52	Solvent free	O_2_	1	8	75	100
6	40, 52	Solvent free	O_2_	3	8	79	96
7	40, 52	Solvent free	Ar	2	8	73	93
8	40, 52	CH_3_CN	O_2_	2	8	71	50
9	40, 52	CH_3_CN/H_2_O	O_2_	2	8	74	90
10	40, 52	H_2_O	O_2_	2	8	69	98

**Table 3:** C–H oxidation reaction of various substrates under optimized conditions

Entry	Substrate	Major product (A)	Time (h)	Yield (%)	Selectivity to A (%)
1^a^	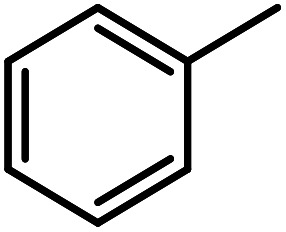	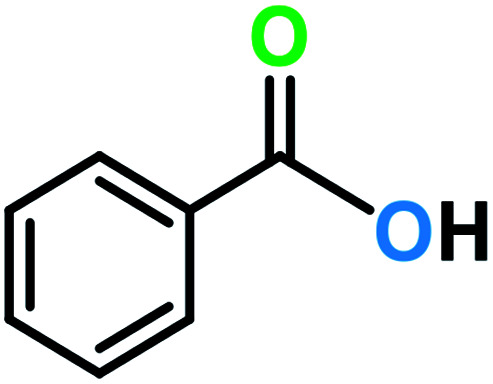	8	99	92
2^a^	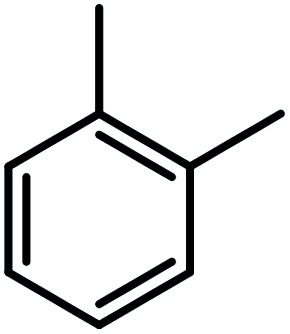	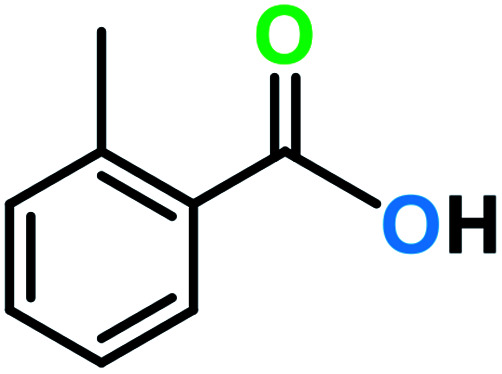	12	90	90
3^a^	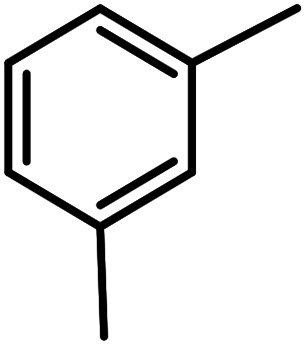	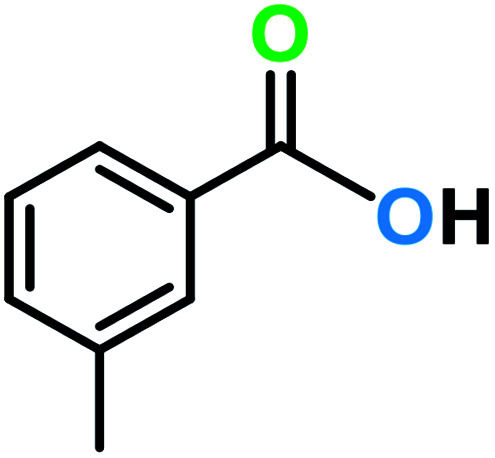	12	87	89
4^a^	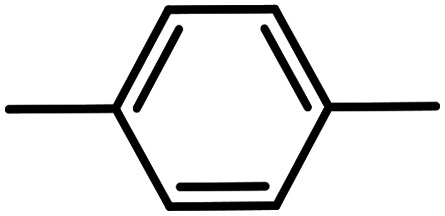	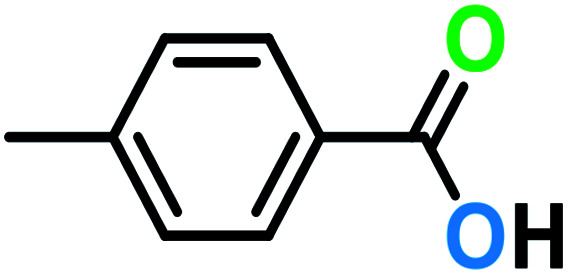	10	>99	97
5^a^	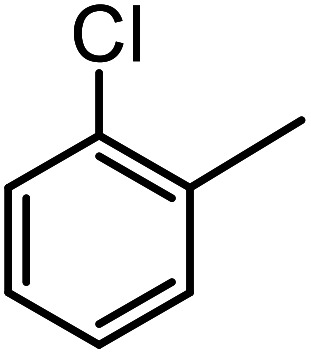	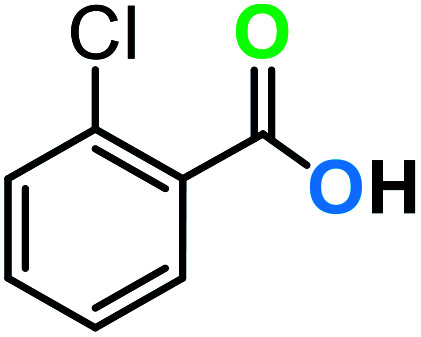	14	72	88
6^a^	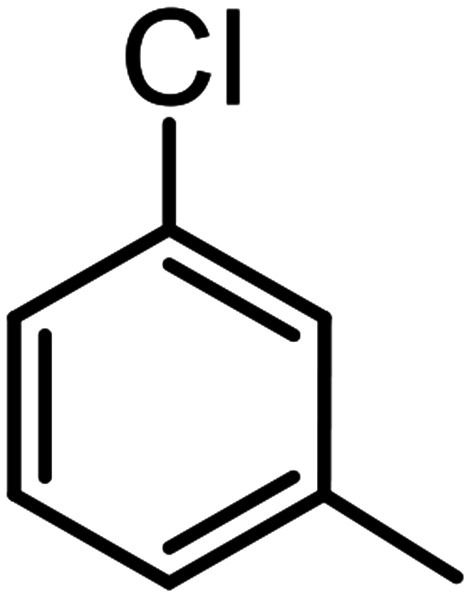	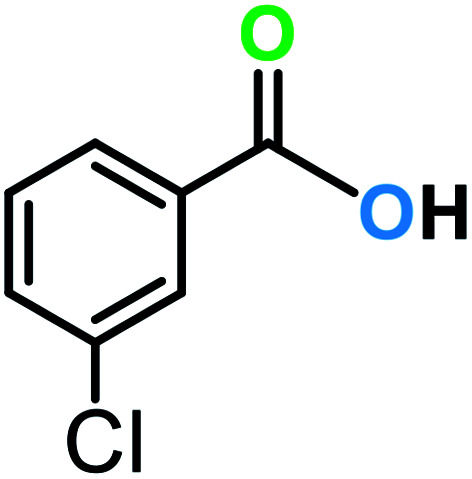	14	76	89
7^a^	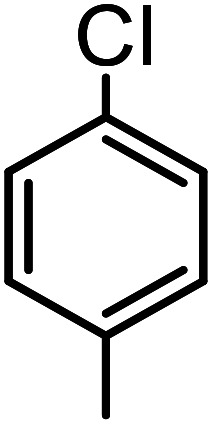	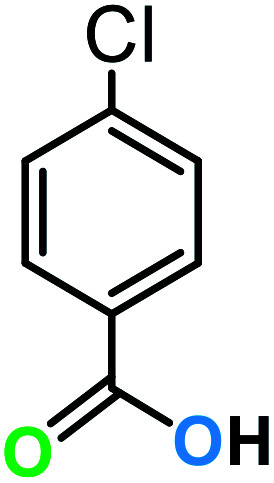	14	82	97
8^a^	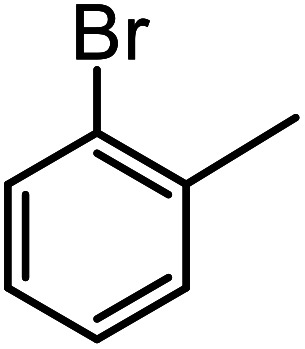	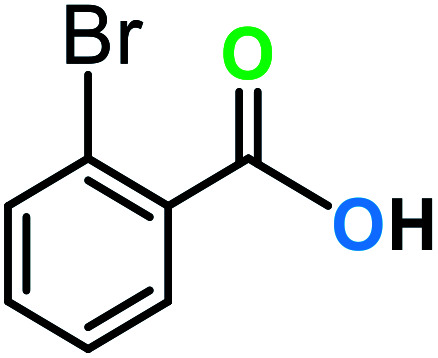	14	90	90
9^a^	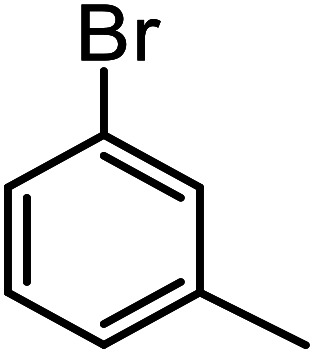	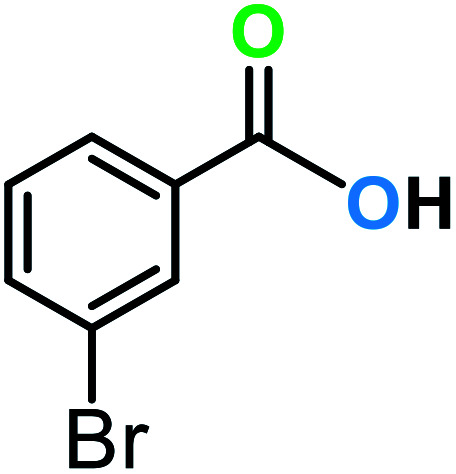	15	81	60
10^a^	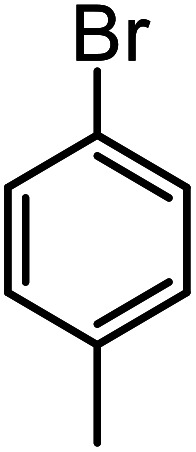	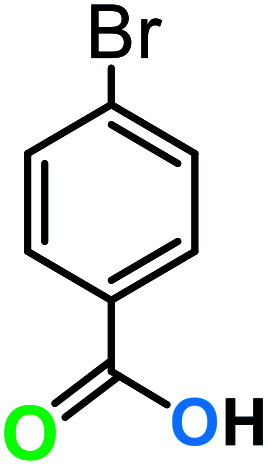	14	99	64
11^a^	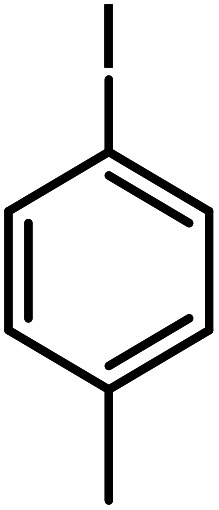	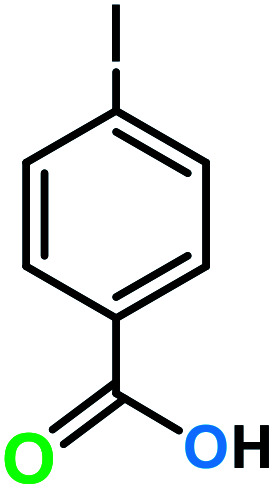	14	>99	100
12^a^	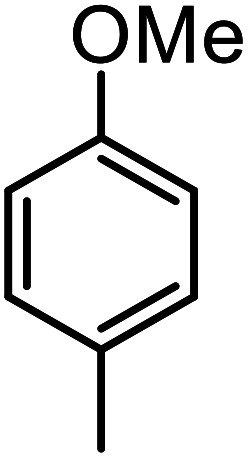	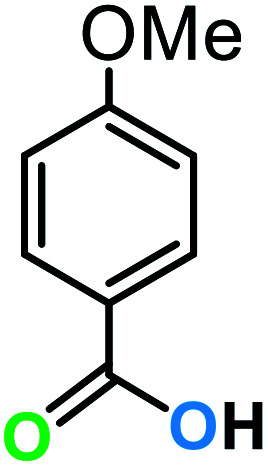	14	91	89
13^a^	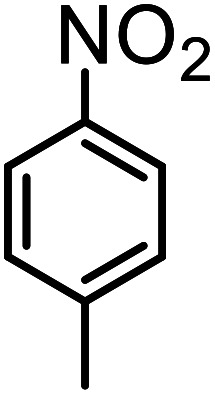	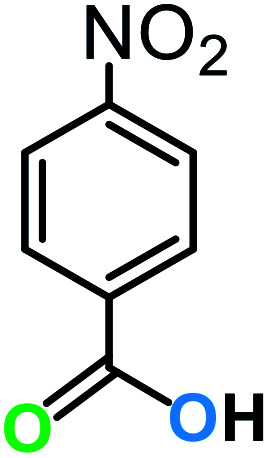	17	60	80
14^b^	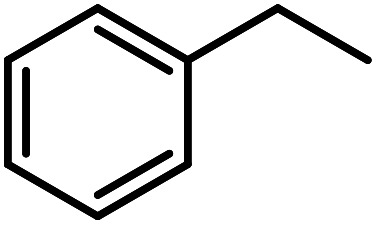	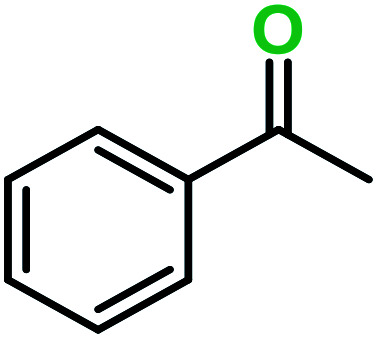	8	>99	96
15^b^^,^^c^	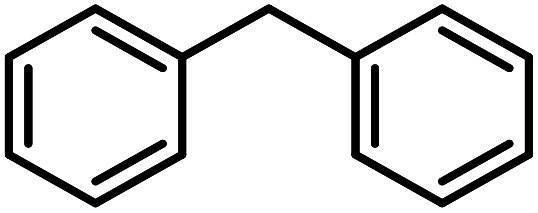	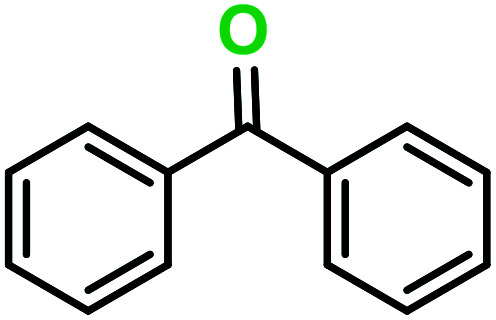	7	81	100
16^b^	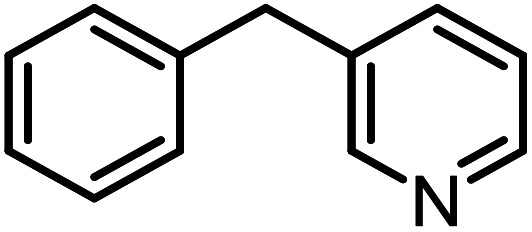	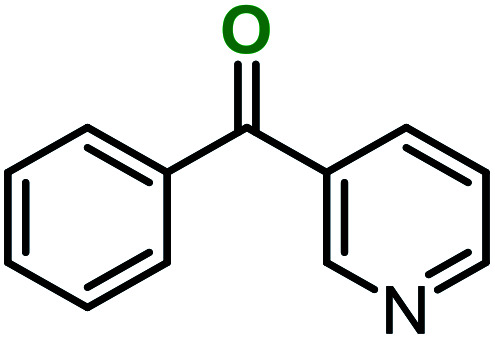	7	>99	>99
17^b^^,^^c^	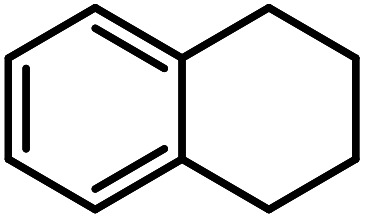	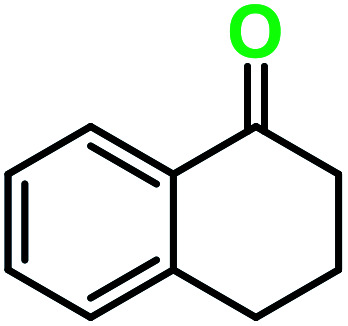	10	72	100
18^d^	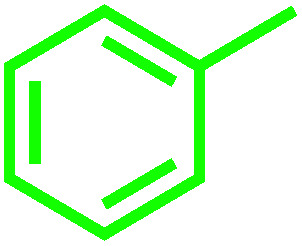	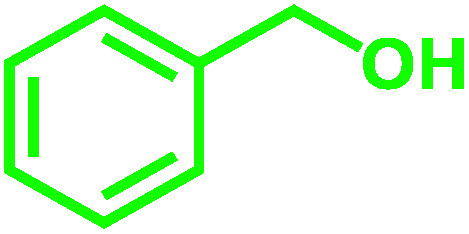	10	99	99
19^d^	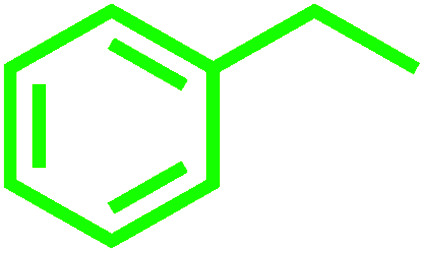	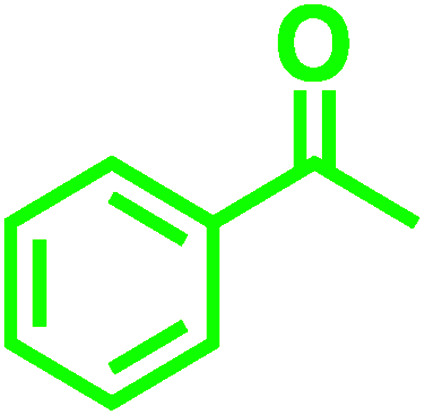	24	99	97

a Reaction parameter: catalyst (40 mg, 52 mol%), substrate (1 mmol), H_2_O (0.5 mL), TBHP (2 equiv. 70% in H_2_O), argon balloon, *T* = RT, yields were determined by isolated yield after purification by chromatographic column.

b Reaction parameter: catalyst (40 mg, 52 mol%), substrate (1 mmol), TBHP (2 equiv. 70% in H_2_O), oxygen balloon, *T* = RT, yields were determined by GC.

c Reaction parameter: TBHP (4 equiv. 70% in H_2_O).

d Reaction parameter: catalyst (40 mg, 52 mol%), substrate (1 mmol), H_2_O (0.5 mL), oxygen balloon, *T* = RT, yields were determined by GC^e^.

e The GC analysis was performed in the presence of anisole as internal standard and the averages of 3 measurements are reported. GC method: 250 °C inlet, 280 °C detector, follow 1 mL min^−1^, oven temperature program: 50 °C for 2 min, 10 °C min^−1^ ramp to 250, and hold at 250 °C for 5 min.

**Scheme 2:**
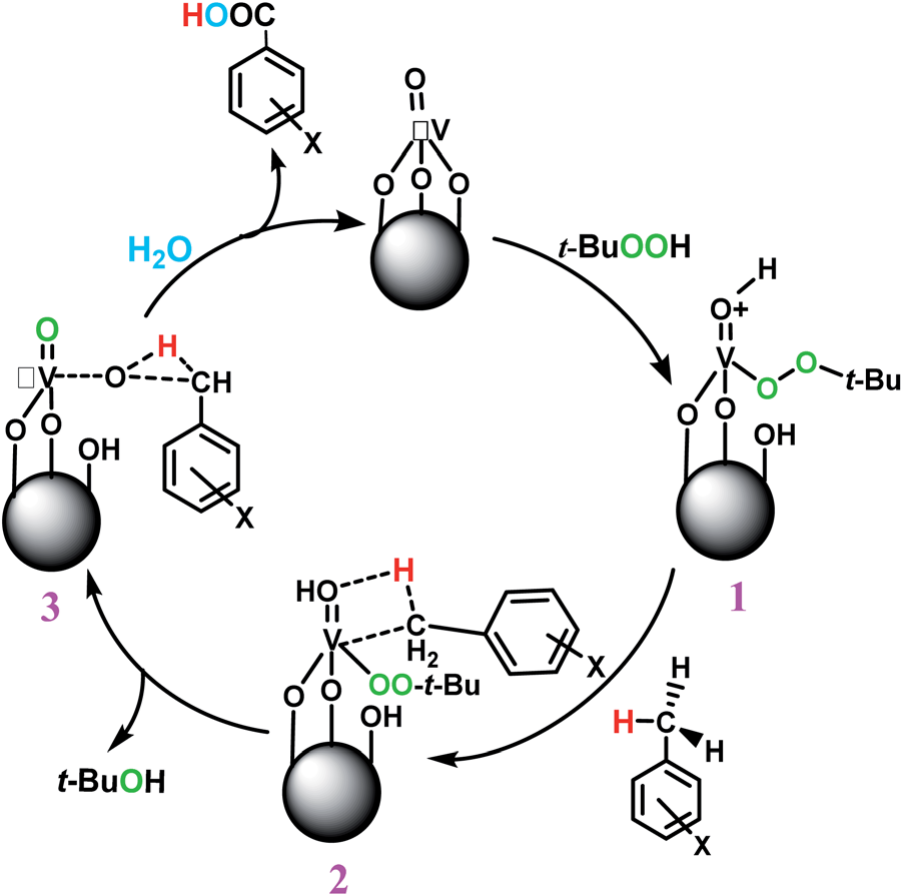
Proposed mechanism for toluene conversion to benzoic acid using TBHP.

**Fig. 3:**
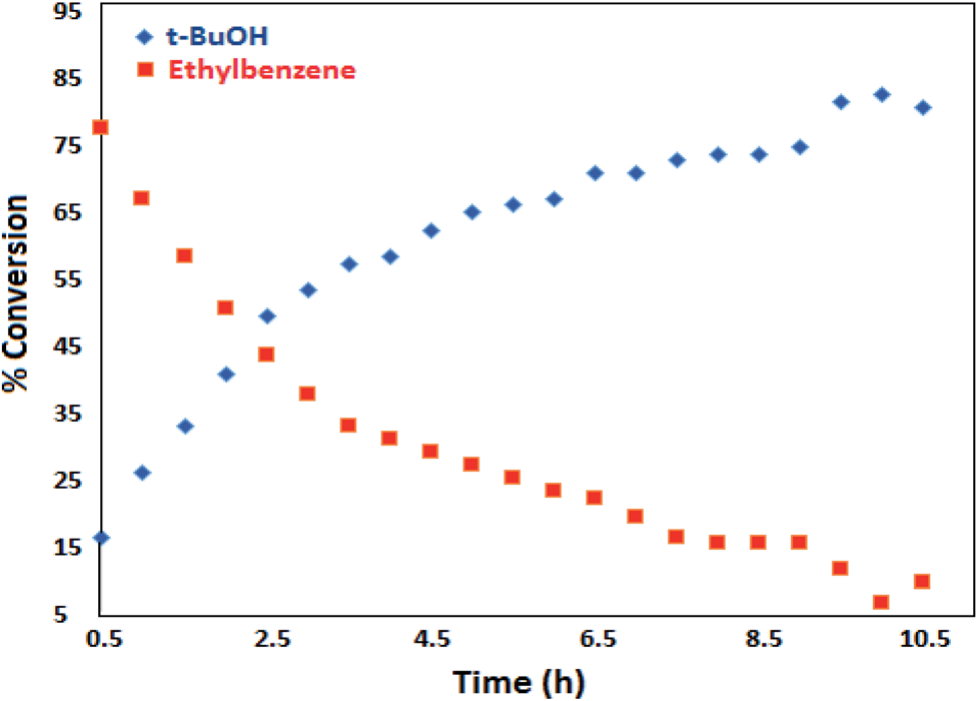
Time monitoring of ethylbenzene consumption and *t*-BuOH production.

The best result was seen with toluene to benzoic acid conversion. The results in Table 3 confirmed that the inductive and resonance effects of *ortho* and *para* substitution can activate the C–H bond more than in *meta* derivatives (Table 3, entries 2–10). In comparison with electron-withdrawing groups, the electron-donating substitutions continued the reactions more effectively (Table 3). According to the observations, in the presence of TBHP, the following mechanism is proposed for C–H activation of toluene (Scheme 2). After addition of TBHP, *t*-BuOOH–vanadyl adduct 1 was formed and the reaction pathway passed through the four-centered transition state of 2 in the presence of toluene. In continuing, *t*-BuOH was released and the oxygen species of 3 formed at the same time. Detection of *t*-BuOH by GC technique is a good indication to confirm this claim. Fig. 3 shows the time monitoring of ethylbenzene consumption and *t*-BuOH production during the reaction process. The presence of H_2_O accelerated the separation of product toward the formation of benzoic acid and improved the selectivity.

**Scheme 3:**
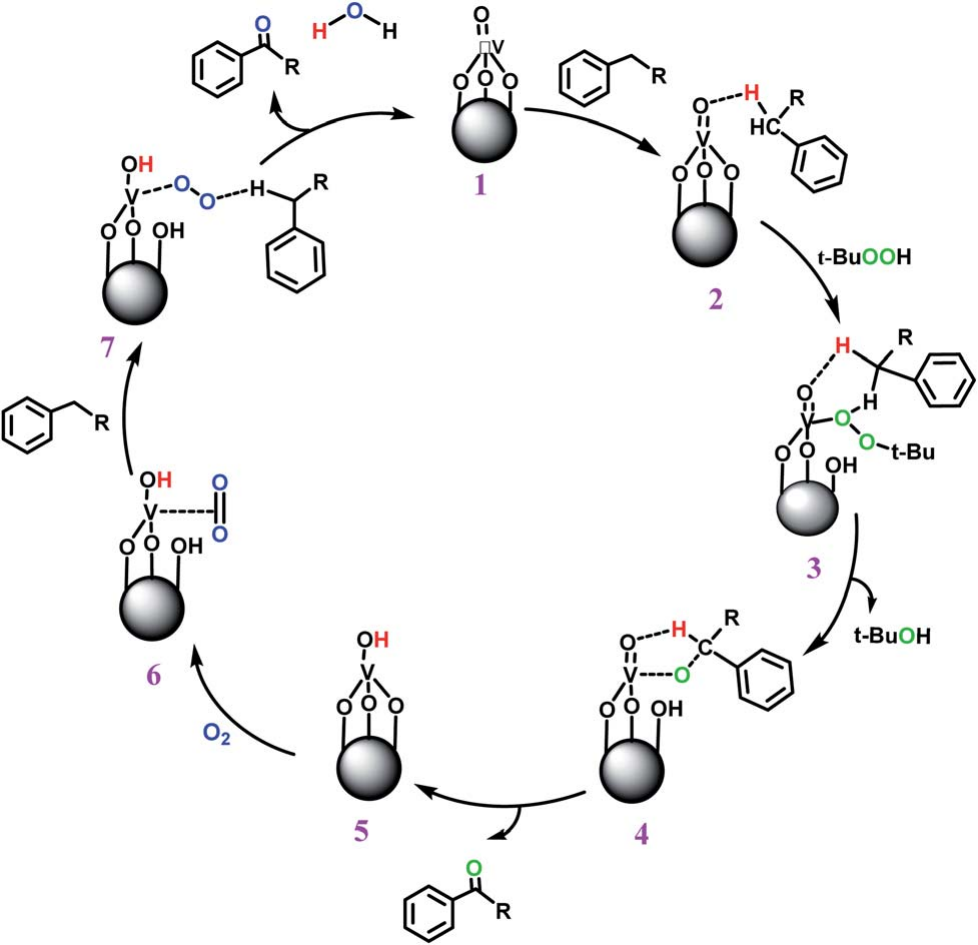
Proposed mechanism for other alkylbenzene oxidation reactions using TBHP.

In another experiment, in the presence of oxygen atmosphere, ethylbenzene was oxidized to acetophenone with high selectivity and full conversion (Table 3, entry 14). As the results show (Table 3, entries 15–17), by increasing the steric effect of substituents on the phenyl ring of ethylbenzene, the conversion percentages were decreased while the selectivity improved remarkably. It seems that molecular oxygen assisted the reaction progress. As the cleavage of C–H bonds in the middle of an alkyl chain is easier than that of terminal ones, aerobic oxidation of ethylbenzene was performed in mild conditions compared to toluene. The proposed mechanism for oxidation of ethylbenzene and other substrates (entries 14–17) is illustrated in Scheme 3. The hydrogen bonding shown in adduct 2 is formed by the approach of alkylbenzene to the catalyst and TBHP which consequently produces the superoxo-vanadium species 3. After O–O bond cleavage, removal of *t*-BuOH (detected by GC monitoring) and H abstraction step, the oxygenated compound was produced. In the absence of O_2_, the observed conversion of ethylbenzene oxidation reaction was 73% (Table 2, entry 7) which suggested that the mechanism was completed aerobically by protonated form of catalyst 5. As in previous steps, the reaction progressed to the final step through superoxo-vanadium intermediate 7 assisted by O_2_ with an accompanying loss of H_2_O and conversion of remaining alkylbenzenes. For further mechanism investigation, the oxidation reactions of toluene and ethylbenzene were carried out in the presence of TEMPO (1 mmol) and identical results strongly indicated that the reaction did not proceed *via* a radical chain pathway.

#### B. Aerobic oxidation of toluene and ethylbenzene

Recently, toluene aerobic oxidation has received a lot of attention and many researches are being carried out in this field according to industrial and economic necessities.^37^ Perhaps performing these types of reactions under totally mild and green conditions was ambitious, but according to the effective potential of the catalyst, this aim was not unrealistic. Surprisingly, at room temperature and in the presence of oxygen-saturated water and oxygen balloon atmosphere, the oxidation of toluene and ethylbenzene took place with excellent conversion and selectivity (Table 3, entries 18, 19). The main product of toluene aerobic oxidation was benzyl alcohol not benzoic acid, while the main product of aerobic oxidation of ethylbenzene was acetophenone as well as TBHP oxidation product.

**Scheme 4:**
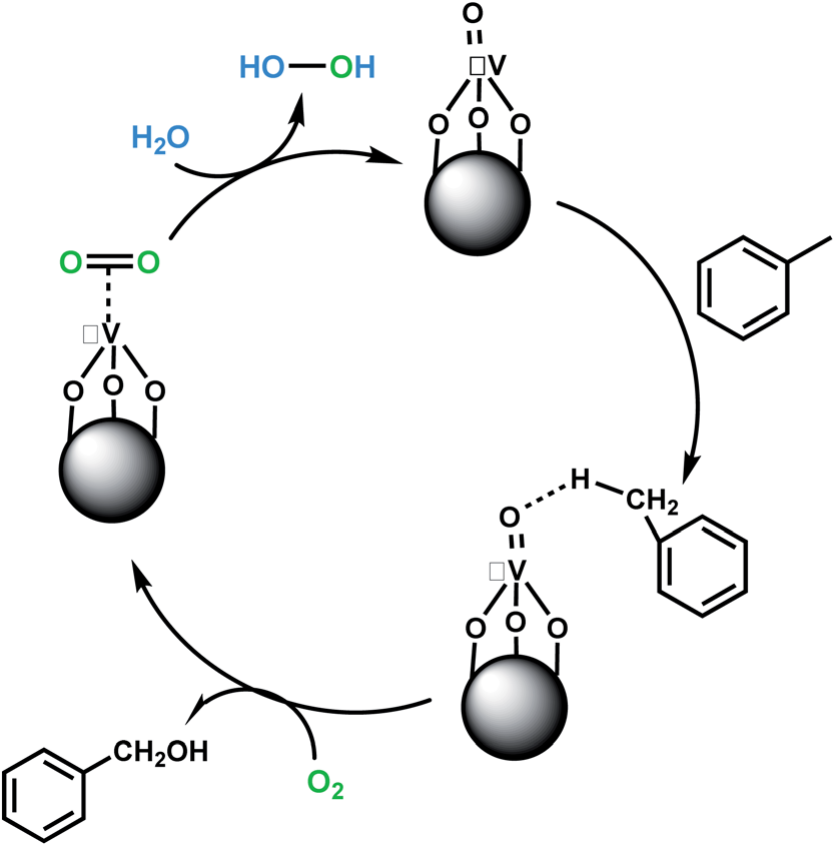
Proposed mechanism for aerobic oxidation of toluene to benzyl alcohol.

The pathway of toluene aerobic oxidation is suggested as shown in Scheme 4. It is expected that the spin-state of O_2_ in the ground state is a triplet while many organic substrates are singlet and their reactions are spin-forbidden. But transition metal complexes could react with triplet O_2_ to produce metal–oxygen intermediates. It was proposed that after hydrogen interaction between toluene and VO^2+^, the molecular oxygen in reaction media facilitated oxygen transfer to toluene. Afterwards, the O=O bond in the formed species was dissociated by water and the catalyst was recovered. The aerobic oxidation of other substrates in Table 3 was not quite so successful and the obtained conversion at around 24 h did not exceed the 15% conversion yield.

**Fig. 4:**
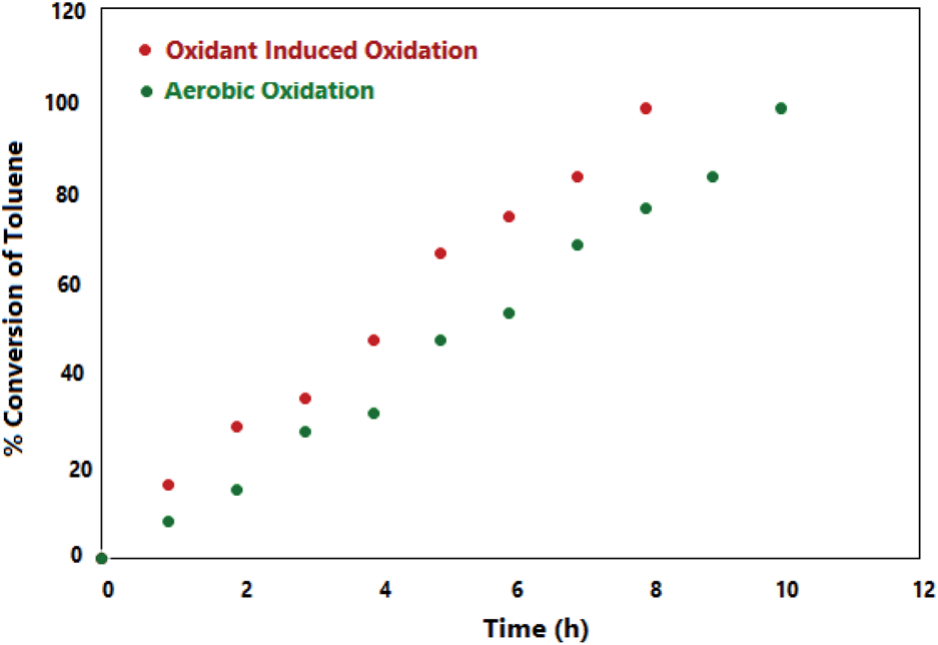
Time monitoring of toluene conversion.

A comparison of time dependence of conversion in both oxygen- and oxidant-induced reactions is clearly shown in Fig. 4.^38^

### Results of hot filtration test and reusability of the catalyst

**Fig. 5:**
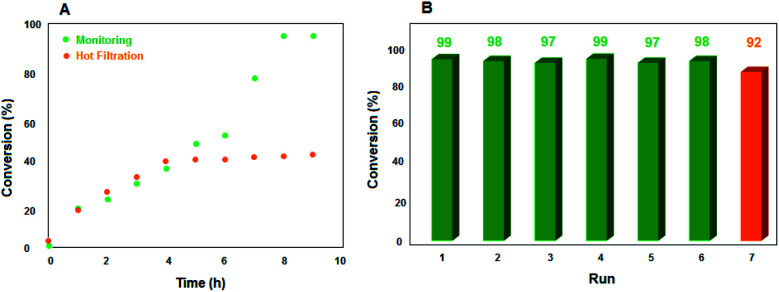
(A) Monitoring and hot filtration test. (B) Recycling of catalyst in ethylbenzene oxidation reaction.

In order to investigate whether VO^2+^ was released from the solid support and to confirm that the reaction pathway proceeded heterogeneously, a hot filtration test for ethylbenzene oxidation reaction under optimized reaction conditions was done. The reaction was monitored by GC in half of the reaction time and the obtained resulting conversion was 42% (Fig. 5A).

According to green chemistry principles, the simple recovery and durability of a catalyst are some of the most important features that also should be considered from an economic viewpoint in industries. To confirm the reusability of the catalyst, it was collected, washed and used several times. After 6 reaction cycles, a 9% reduction of conversion was seen (Fig. 5B). FETEM observation confirmed the maintenance of the morphology of the recovered catalyst (Fig. 1E). Furthermore, ICP analysis determined that the leached amount of VO^2+^ after completion of cycle 7 was negligible (<1 mol%). Therefore, the stability and reusability of the catalyst were appropriate for its use in important reactions.

Particularly, reports of C–H bond oxidation reactions (especially for toluene and its derivatives) using heterogeneous catalysts at room temperature are rare and a comparison of this work with recent reports indicates distinctly the efficacy of this catalyst (Table 4).

**Table 4:** Comparison of this work with some recent publications for catalytic oxidation reactions of toluene

Entry	Catalyst	Reaction conditions	Conv. (%)	Major product
1 (ref. 29)	MnWO_4_ nanobars (0.01 g, 5 wt%)	Toluene (0.2 mL), CH_3_CN (8 mL), H_2_O_2_ (3 equiv.), *T* = 80 °C, time = 24 h	60	Benzaldehyde
2 (ref. 30)	Mn_0.3_Zr_0.7_O_2_	Toluene (1000 ppm), 20% O_2_ balanced by N_2_, total flow rate = 50 mL min^−1^, weight hourly space velocity = 60 000 mL g_cat_^−1^ h^−1^, *T* = 235 °C, in a fixed-bed quartz tubular reactor	90	Maleic anhydride
3 (ref. 31)	Cu functionalized nano-crystalline ZSM-5 (1 g, 0.4 wt%)	Toluene (25 mL), deionized water (25 mL), H_2_O_2_ (25 mL), *T* = 180 °C, time = 4 h, in a PARR autoclave reactor, N_2_ gas pressure = 5.0 bar	96	Benzoic acid
4 (ref. 32)	Pd–Ag@CeO_2_ (100 mg)	Toluene (500 ppm), 20% O_2_ balanced with N_2_, *T* = 88 °C, total gas flow rate = 33.3 mL min^−1^, visible light intensity = 160 mW cm^−2^	50	Not identified
5 (ref. 33)	Polystyrene grafted vanadium Schiff base complex (30 mg, 6.58 wt%)	Toluene (5 mmol), CH_3_CN (10 mL), H_2_O_2_ (15 mmol), *T* = 65 °C, time = 6 h	79	Benzaldehyde
6 (ref. 34)	Pt_28_ subnanocatalyst (10 mg, 0.216 wt%)	Toluene (2 mL), O_2_ (1 MPa), *T* = 160 °C, time = 15 h, in an autoclave reactor	1000 μmol product	Benzoic acid
7 (ref. 35)	MnTPPCl (1.0 × 10^−3^ mmol)	Toluene (5 mmol), cyclohexene (3 mmol), CH_3_CN (10 mL), O_2_ (1.2 MPa), *T* = 160 °C, time = 4 h	13	Benzoic acid
8 (ref. 36)	Fe_3_O_4_@SiO_2_-APTES-MnL^GDC^ (40 mg, 2 mol%)	Toluene (1 mmol), solvent free, TBHP (4 equiv., 70%), *T* = 60 °C, time = 20 h	71	Benzoic acid
**This work**	**VO** ^**2+**^ **@SiO**_**2**_ **@Fe**_**3**_ **O**_**4**_ **(40 mg, 52 mol%)**	**Toluene (1 mmol), H**_**2**_ **O (0.5 mL), O**_**2**_ **balloon, *T* = RT, time = 10 h**	**99**	**Benzyl alcohol**
		**Toluene (1 mmol), H**_**2**_ **O (0.5 mL), Ar, TBHP (2 equiv., 70%), *T* = RT, time = 8 h**	**99**	**Benzoic acid**

## Conclusions

In conclusion, to convert aromatic hydrocarbons especially toluene and xylenes which are typical petroleum contaminants to useful compounds for other industries, we have introduced a highly efficient and simple earth-abundant vanadyl-based magnetic nano-catalyst. We applied a green solvent or no solvent and relatively green reagents with the minimum organic material usage under very mild reaction conditions of room temperature to produce important oxygenated compounds through C–H activation reaction. Aerobic and totally green oxidation reactions of toluene and ethylbenzene demonstrated that immobilization of VO^2+^ on silica-coated Fe_3_O_4_ nanoparticles made it an excellent catalyst which can be used in important industrial reactions with minimal energy consumption and pollution. Furthermore, the simplicity of the catalyst structure and good capability of the oxo vanadium(iv) cation in the oxygen transfer process as well as recoverability and reusability of the nanomagnet make the catalyst efficient and sustainable. All these points show that we are standing at the closest point to the dream of green chemistry for the alkylbenzene C–H activation process.

## Experimental section

### Materials and methods

All reagents and solvents were purchased from Merck and Sigma Aldrich sources and used without further purification. FTIR spectra from KBr pellets of the compounds were recorded using a Shimadzu FT-IR-8300 spectrophotometer. The morphological topographies of particles were evaluated using FETEM (JEOL, JEM-2100F, 200 kV). FESEM analysis and EDX were performed with a Tescan Mira (II) at an acceleration voltage of 15 kV. The X-ray powder patterns were obtained using a Philips PW1730 (step size: 0.05, time per step: 1 s). The magnetic properties of the catalyst were measured using VSM with homemade vibrating sample magnetometer apparatus (Meghnatis Daghigh Kavir Company, Iran) at RT from −10 000 to +10 000 Oe. The electronic states of the powders were investigated using XPS (Multilab 2000, Thermo Scientific, Al Kα radiation). Furthermore, the reactions were followed using thin layer chromatography (TLC) and gas chromatography (GC, Agilent gas chromatograph model 7890A) with an HP-1 methyl siloxane column (30 m in length × 320 μm × 0.25 μm) using a flame ionization detector.

### Synthesis of magnetic nanoparticles (SiO_2_@Fe_3_O_4_)

Magnetic nanoparticles (Fe_3_O_4_) were prepared using a co-precipitation method according to a literature procedure.^39^ 5.2 g of FeCl_3_·6H_2_O and 2.8 g of FeSO_4_ were dissolved in 25 mL of deionized water, followed by adding 0.85 mL of HCl (37%) under Ar gas. The resulting solution was added dropwise into 25 mL of 1.5 mol L^−1^ NaOH solution with mechanical stirring under argon atmosphere at 80 °C. The produced Fe_3_O_4_ nanoparticles were magnetically separated and rinsed three times with water and ethanol. According to the literature,^40^ Fe_3_O_4_ nanoparticles (0.5 g) were dispersed in a mixture of ethanol (50 mL), water (5 mL), and tetraethoxysilane (0.20 mL). Afterward, 5 mL NaOH (10 wt%) was added dropwise and the reaction was allowed to proceed under mechanical stirring. After stirring at room temperature for 30 min, the SiO_2_@Fe_3_O_4_ particles were separated using a magnetic field and washed with ethanol and water three times and dried under vacuum at 80 °C for 10 h.

### Synthesis of catalyst

The silica-coated Fe_3_O_4_ nanoparticles were dried at 80 °C overnight. Incorporation of VO^2+^ species was carried out in an argon-filled glove box. To a stirring suspension of SiO_2_@Fe_3_O_4_ (1 g) in dry dichloromethane (40 mL), 0.2 mL of VOCl_3_ was added dropwise and the reaction mixture continued stirring for 3 days at room temperature. The resulting material was separated from the reaction mixture using an external magnet and washed three times with dichloromethane, acetone and ethanol. The final light-brown to green material was dried at 80 °C for 12 h.

### General procedure for oxidant-mediated and aerobic oxidation of alkylbenzenes

In a 5 mL round-bottom flask, a mixture of 40 mg catalyst and 1 mmol toluene derivative was stirred in the presence of water (0.5 mL) and 2 mmol TBHP as oxidant was added. The reaction was carried out under argon atmosphere and at room temperature. Other alkylbenzene oxidation processes were carried out under oxygen atmosphere and solvent-free reaction conditions.

In order to perform aerobic oxidation reactions, the catalyst (40 mg, 52 mol%) and substrate (1 mmol) were stirred in the presence of 0.5 mL water (saturated with oxygen for 15 minutes), under oxygen balloon atmosphere at room temperature.

The conversion and selectivity were monitored by GC or TLC (Table 3, entries 5–13) and, after completion of the reaction, the mixture was extracted with ethyl acetate and then the catalyst was recovered using an external magnet. The products were collected, purified and identified by ^1^H NMR spectroscopy.

### Hot filtration test and reusability of the catalyst

A hot filtration test for ethylbenzene oxidation reaction under optimized reaction conditions was done. For this purpose, after 4 h, the catalyst was separated easily using an external magnet and the reaction was allowed to go on for a further 5 h. The reaction process was monitored by GC and the obtained resulting conversion was only 42% (Fig. 5A). To confirm the reusability of the catalyst, after the completion of the first ethylbenzene oxidation reaction cycle, the catalyst was removed from the reaction mixture magnetically, washed several times with ethyl acetate and ethanol, dried at 70 °C and used in a new reaction cycle. This was repeated for 6 reaction cycles. FETEM and ICP analysis determined the VO^2+^ leaching after completion of cycle 7.

## Conflicts of interest

The authors declare no conflict of interest.

## Supplementary Material

RA-010-D0RA03483E-s001
