# Supplementary material for: Catalytic C–H aerobic and oxidant-induced oxidation of alkylbenzenes (including toluene derivatives) over VO2+ immobilized on core–shell Fe3O4@SiO2 at room temperature in water
Source: RSC Adv. 2020 Jun 22;10(40):23543–53. doi: 10.1039/d0ra03483e (PMC9055093; doi:10.1039/d0ra03483e)

Electronic Supplementary Material (ESI) for RSC Advances.
This journal is © The Royal Society of Chemistry 2020

VO^{2+}@SiO_2@Fe_3O_4 Core/Shell-mediated C-H aerobic and oxidant induced oxidation of alkylbenzenes (including toluene derivatives) at room temperature in water

Pegah Mohammadpour[a] and Elham Safaei*[b]

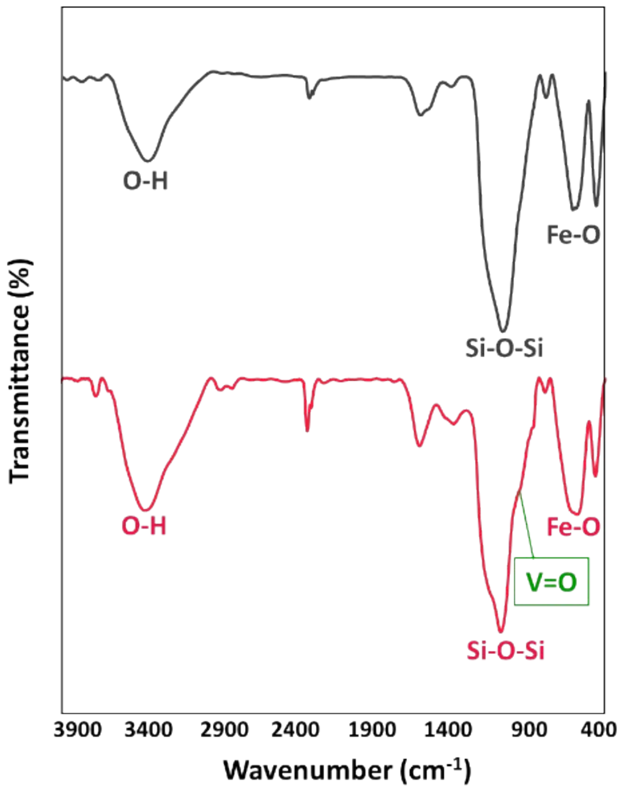

Figure S1. FTIR spectra of A) SiO_2@Fe_3O_4 and B) VO^{2+}@SiO_2@Fe_3O_4.

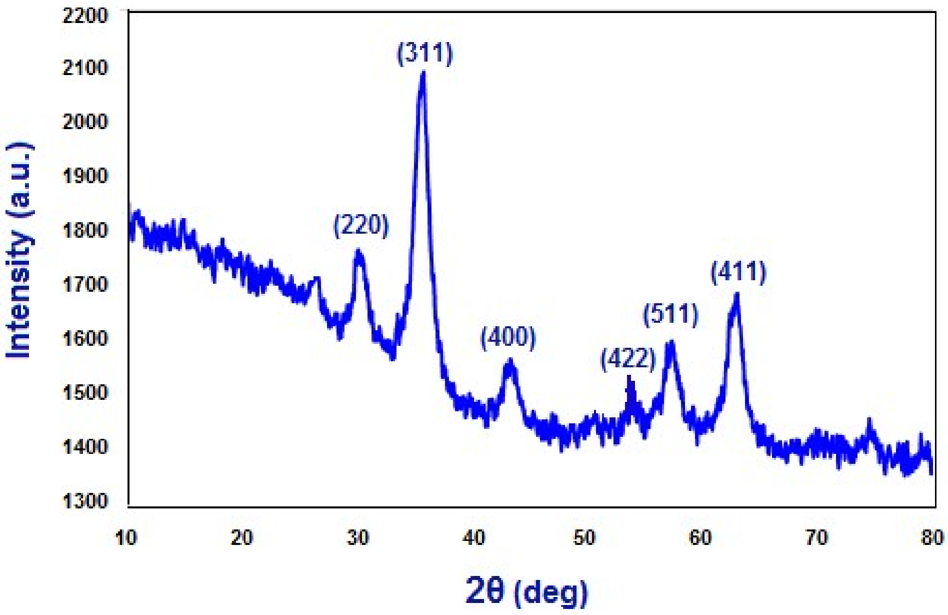

Figure S2. XRD pattern of VO^{2+}@SiO_2@Fe_3O_4.

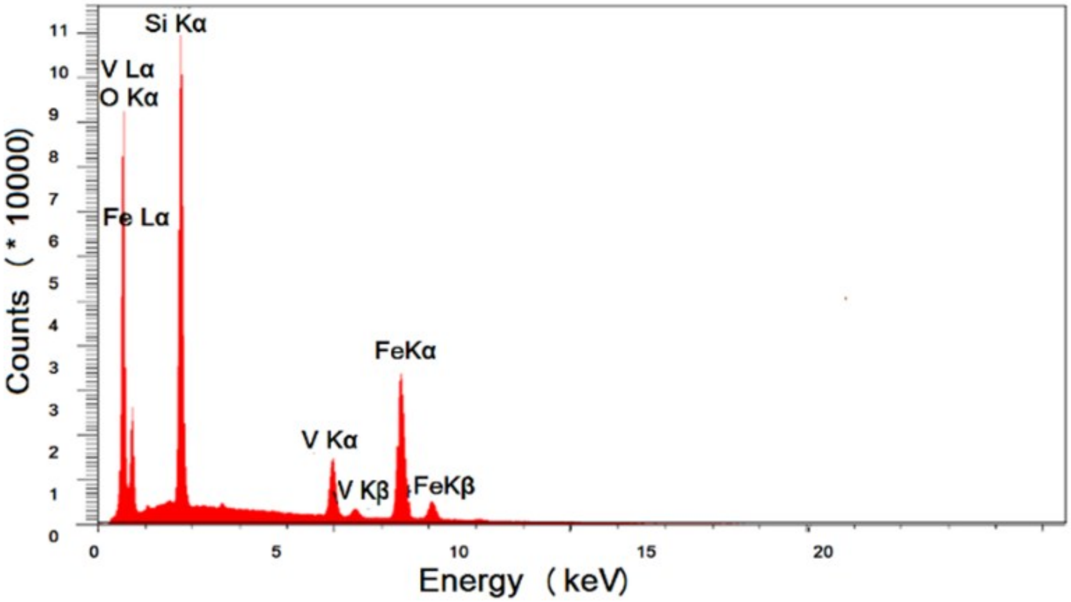

Figure S3. Energy-dispersive X-ray spectroscopy of $VO^{2+}@SiO_2@Fe_3O_4$.

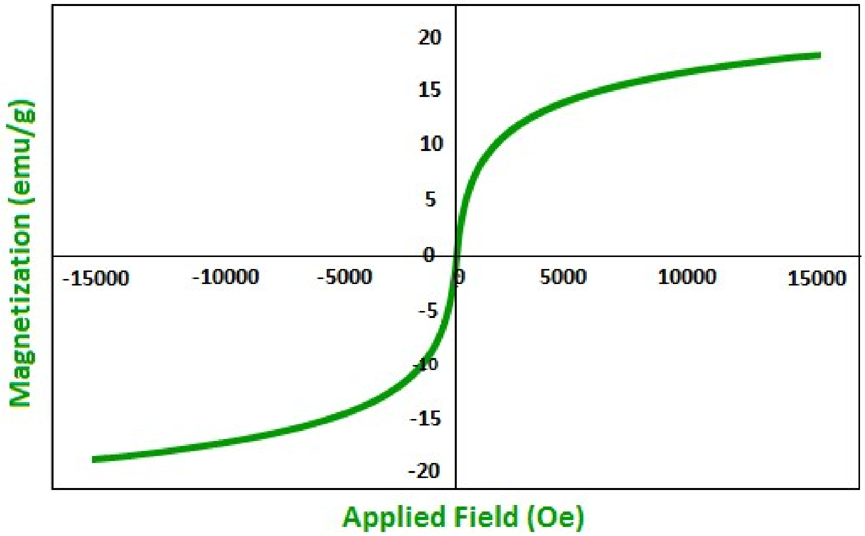

Figure 5. Magnetization curve of $VO^{2+}@SiO_2@Fe_3O_4$.

^{1}H NMR spectra for all synthesized compounds

1. Benzoic acid

^{1}H NMR (400 MHz, d_6-DMSO): δ (ppm) = 12.97 (s, 1H), 7.97 (d, J = 8 Hz, 2H), 7.64 (t, J= 8 Hz, 1H), 7.52 (t, J= 8 Hz, 2H).

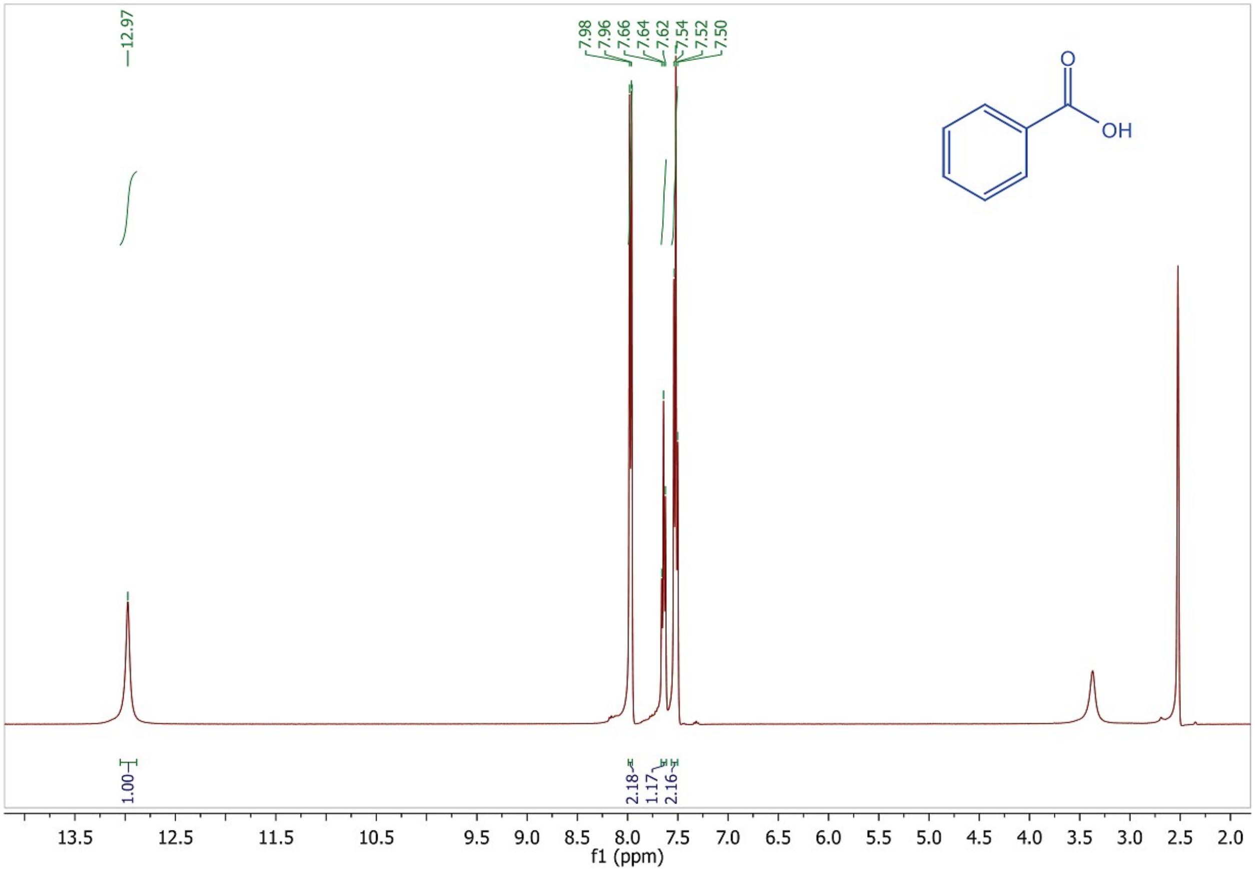

2. 2-Methylbenzoic acid

^{1}H NMR (400 MHz, d_{6}-DMSO): δ (ppm) = 13.14 (s, 1H), 7.84-7.31 (m, 4H), 2.54 (s, 1H).

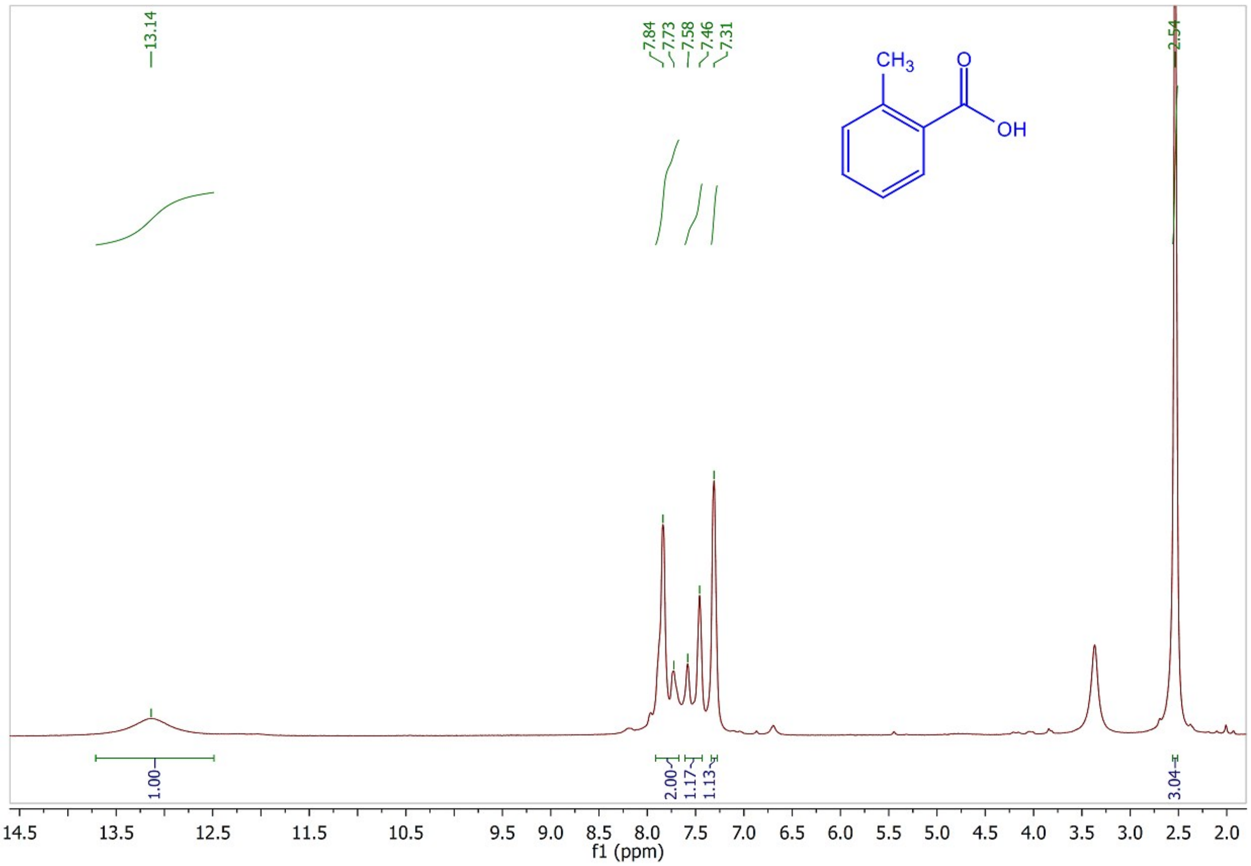

3. 3-Methylbenzoic acid

^{1}H NMR (400 MHz, d_6-DMSO): δ (ppm) = 13.12 (s, 1H), 8.51- 7.45 (m, 4H), 2.39 (s, 3H).

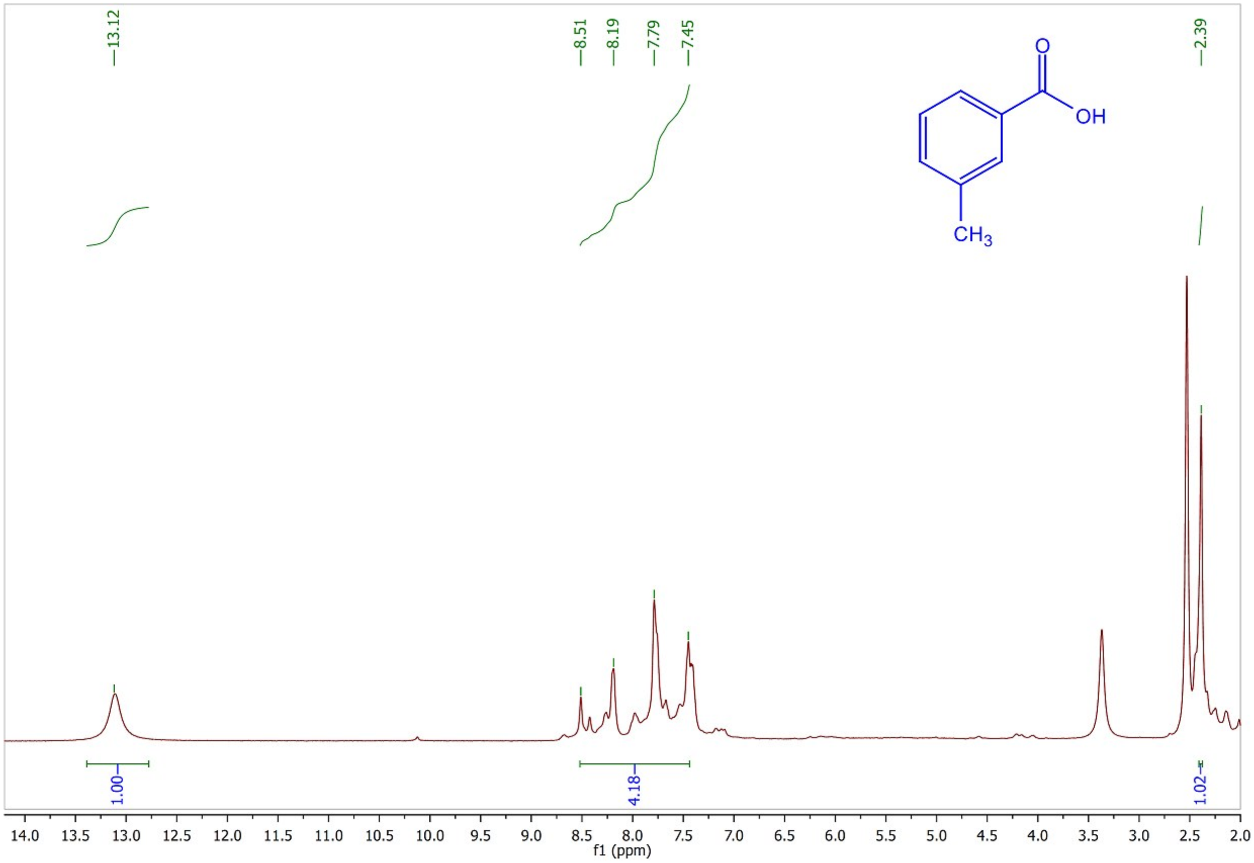

4. 4-Methylbenzoic acid

^{1}H NMR (400 MHz, d_6-DMSO): δ (ppm) = 12.80 (s, 1H), 7.86 (d, *J* = 8 Hz, 2H), 7.31 (d, *J*= 8 Hz, 2H), 2.38 (s, 3H).

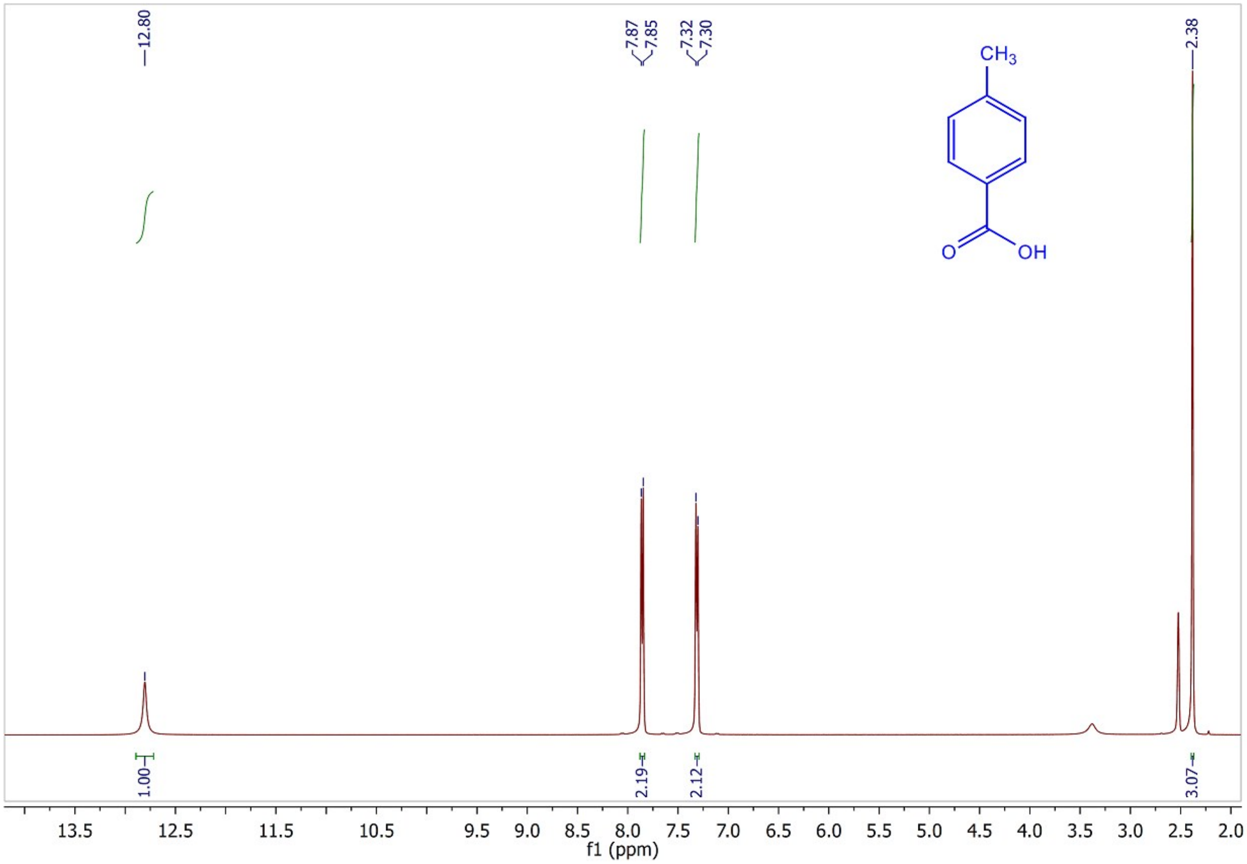

5. 2-Chlorobenzoic acid

^{1}H NMR (400 MHz, d_6-DMSO): δ (ppm) = 13.35 (s, 1H), 7.91-7.92 (m, 2H), 7.71 (d, *J*= 8 Hz, 1H), 7.55 (t, *J*= 8 Hz, 1H).

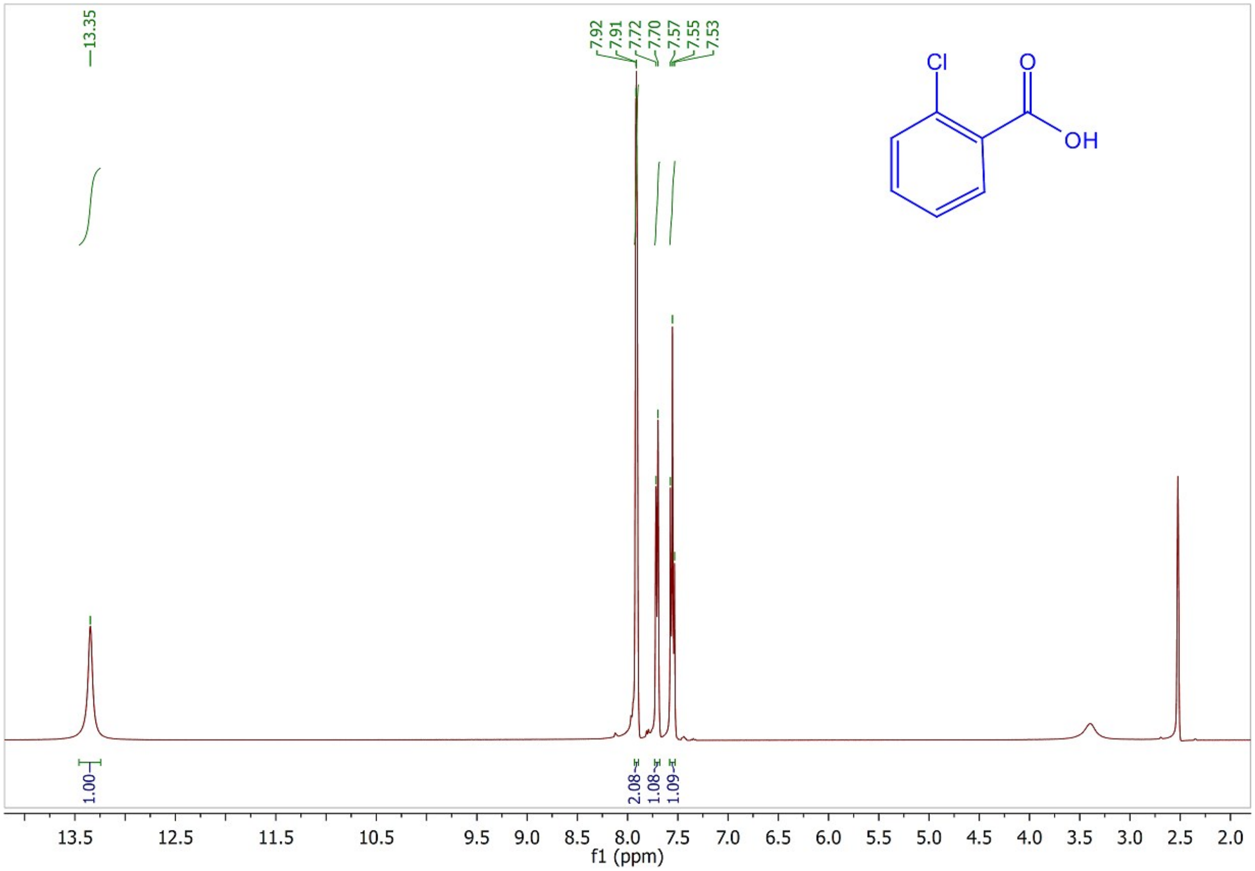

6. 3-Chlorobenzoic acid

^{1}H NMR (400 MHz, d_6-DMSO): δ (ppm) = 13.40 (s, 1H), 7.80 (d, J= 8 Hz, 1H), 7.56 (s, 2H), 7.45 (t, *J*= 8 Hz, 1H).

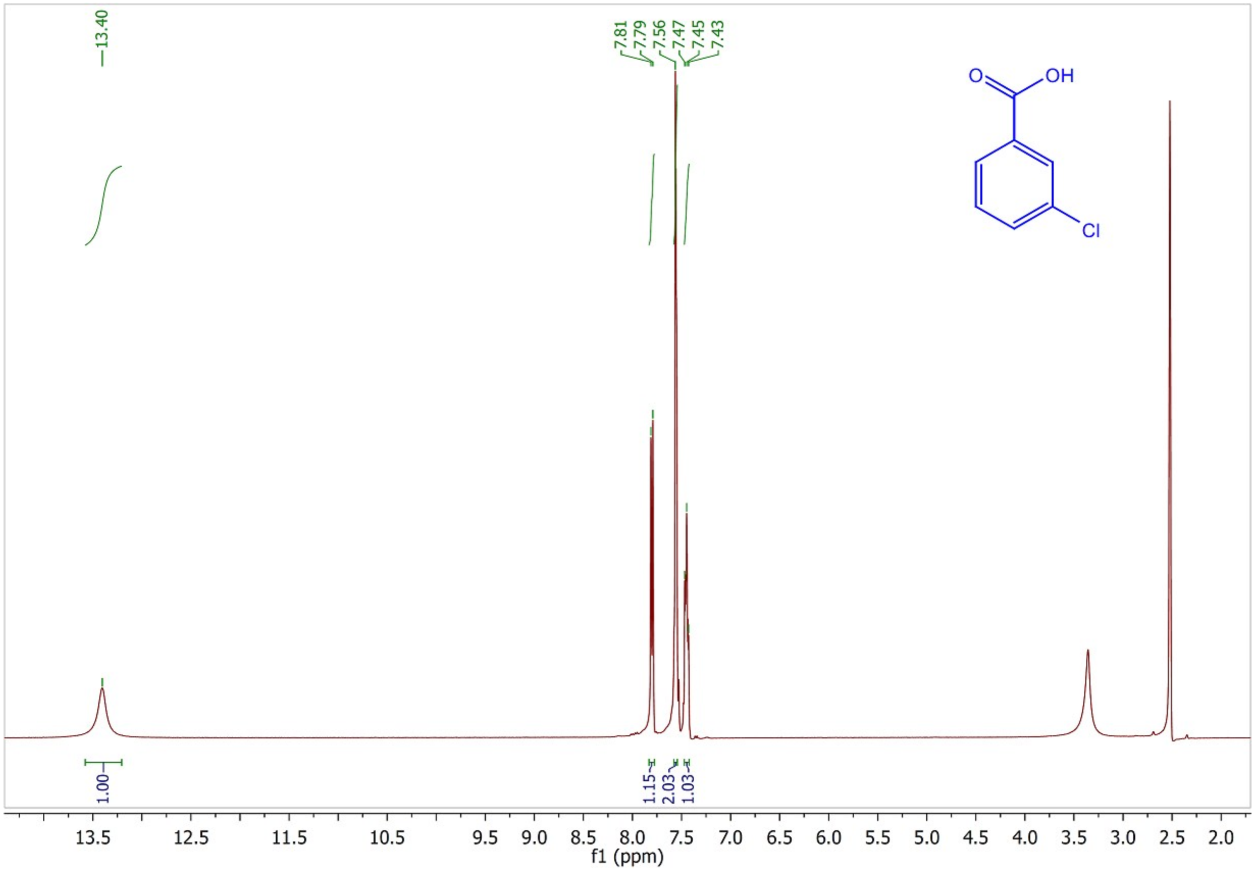

7. 4-Chlorobenzoic acid

^{1}H NMR (400 MHz, d_6-DMSO): δ (ppm) = 13.20 (s, 1H), 7.96 (d, *J*= 8 Hz, 2H), 7.59 (d, *J*= 8 Hz, 2H).

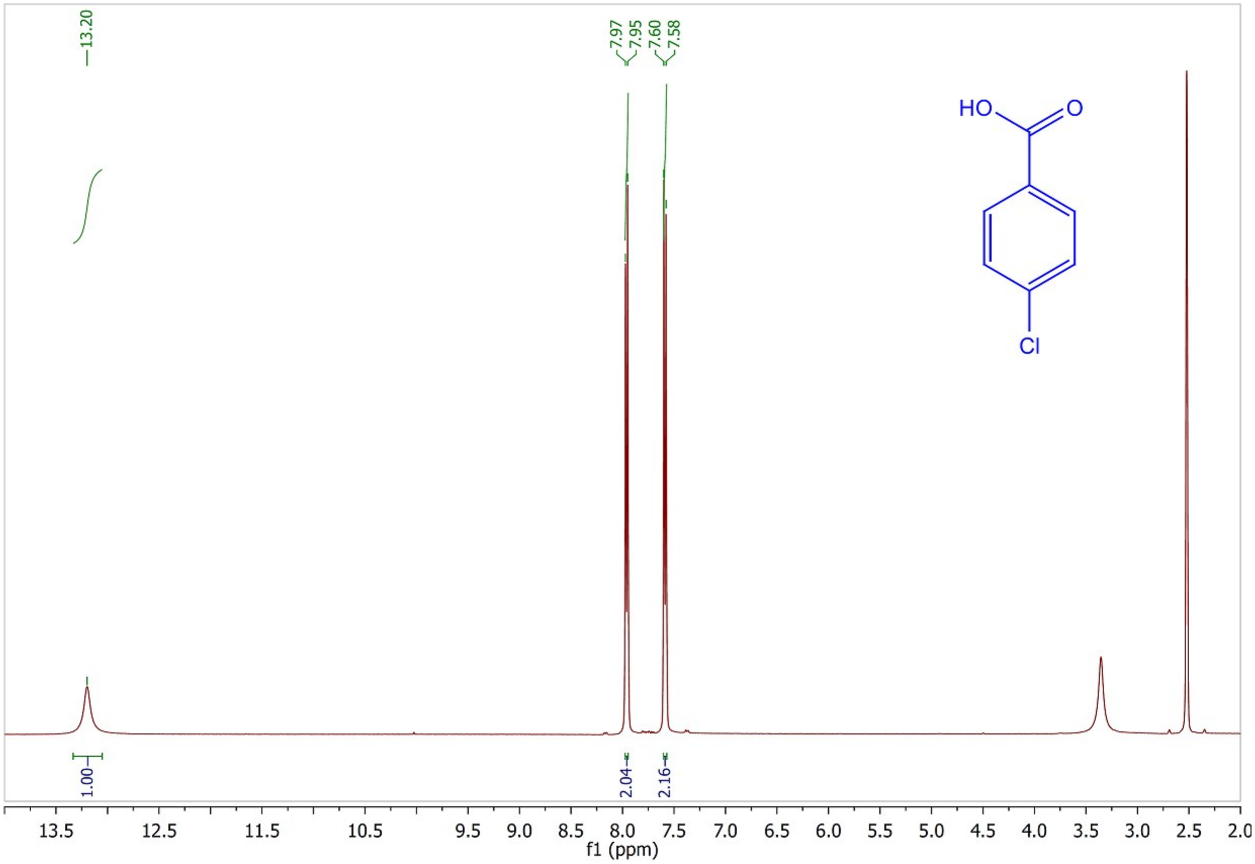

8. 2-Bromobenzoic acid

^{1}H NMR (400 MHz, *d*$_6$-DMSO): δ (ppm) = 13.41 (s, 1H), 7.77- 7.71 (m, 2H), 7.49- 7.40 (m, 2H).

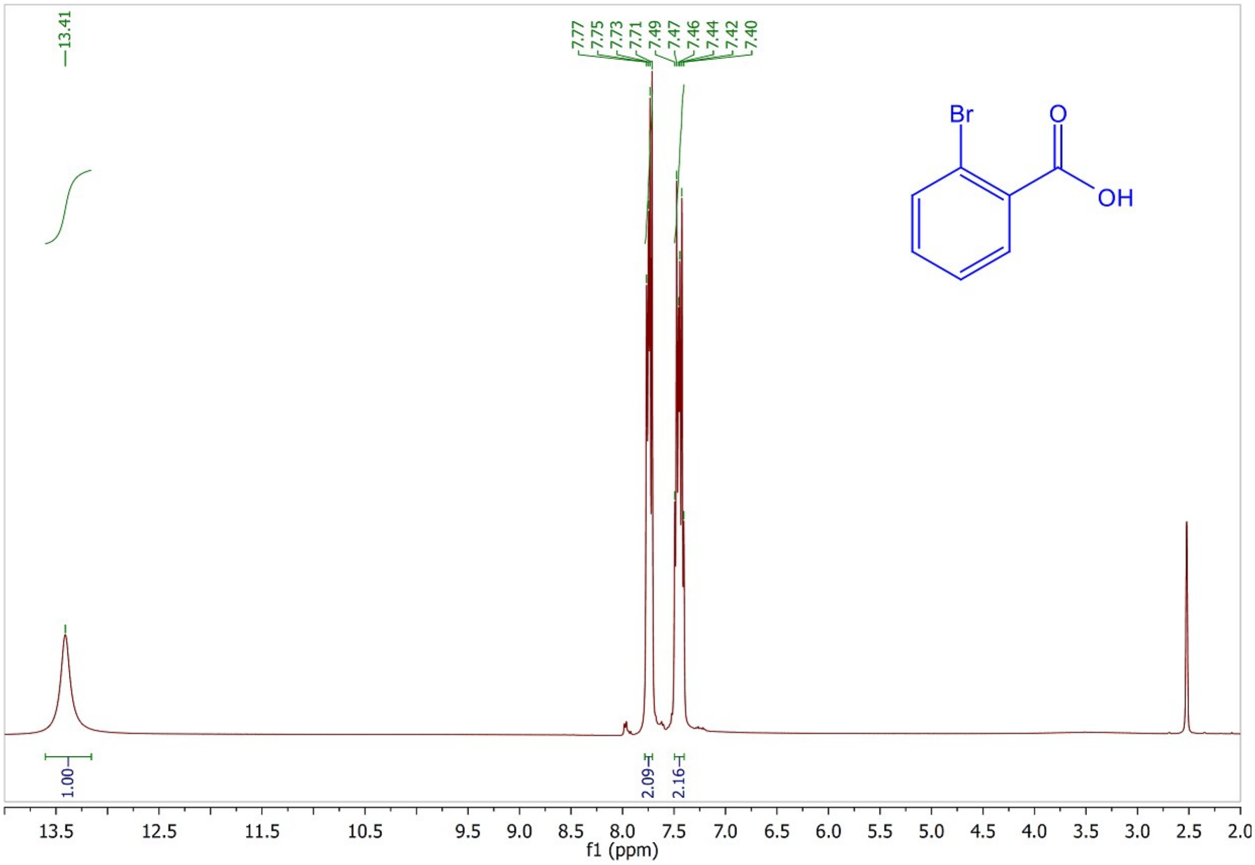

9. 3-Bromobenzoic acid

^{1}H NMR (400 MHz, d_6-DMSO): δ (ppm) = 13.36 (s, 1H), 8.06 (s, 1H), 7.95 (d, *J*= 8 Hz, 1H), 7.85 (d, *J*= 8 Hz, 1H), 7.49 (t, *J*= 8 Hz, 1H).

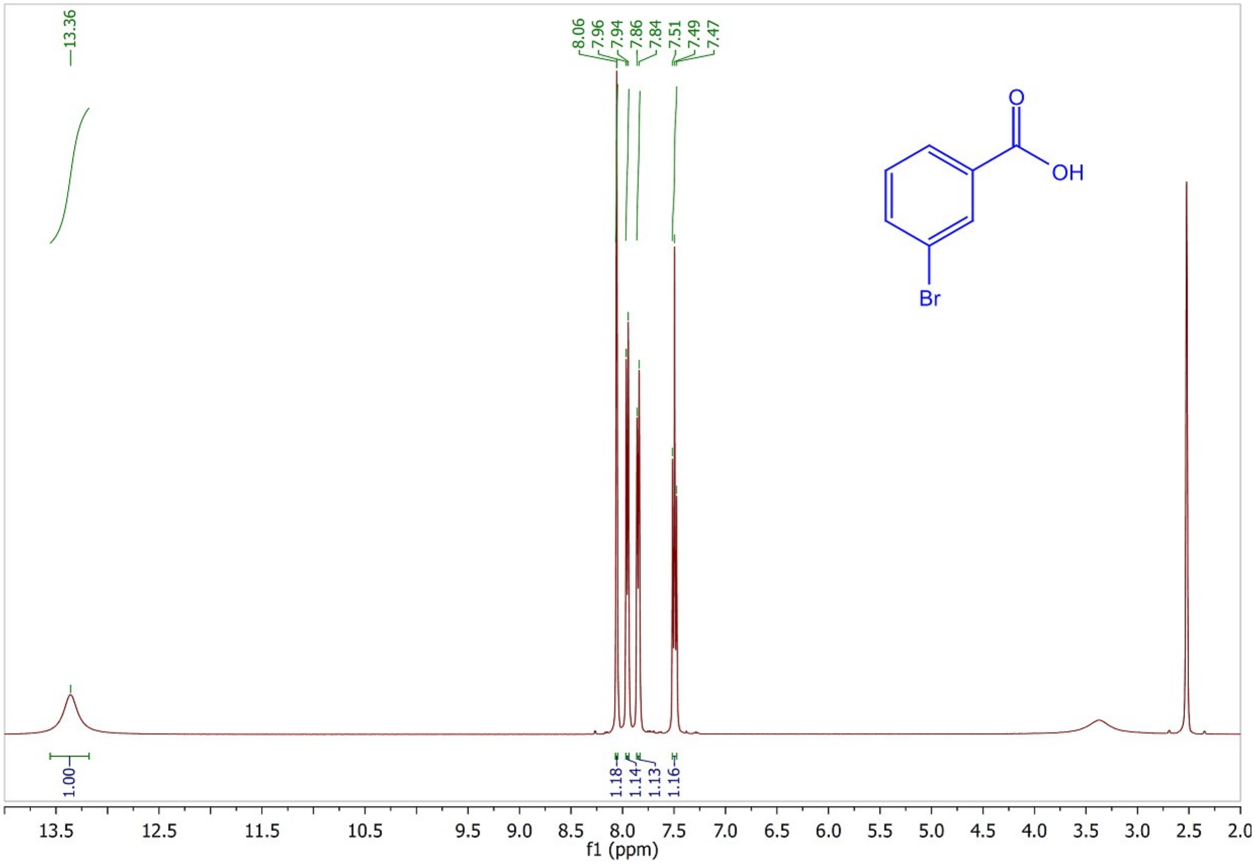

10. 4-Bromobenzoic acid

^{1}H NMR (400 MHz, d_6-DMSO): δ (ppm) = 13.22 (s, 1H), 7.88 (d, *J*= 8 Hz, 2H), 7.73 (d, *J*= 8 Hz, 2H).

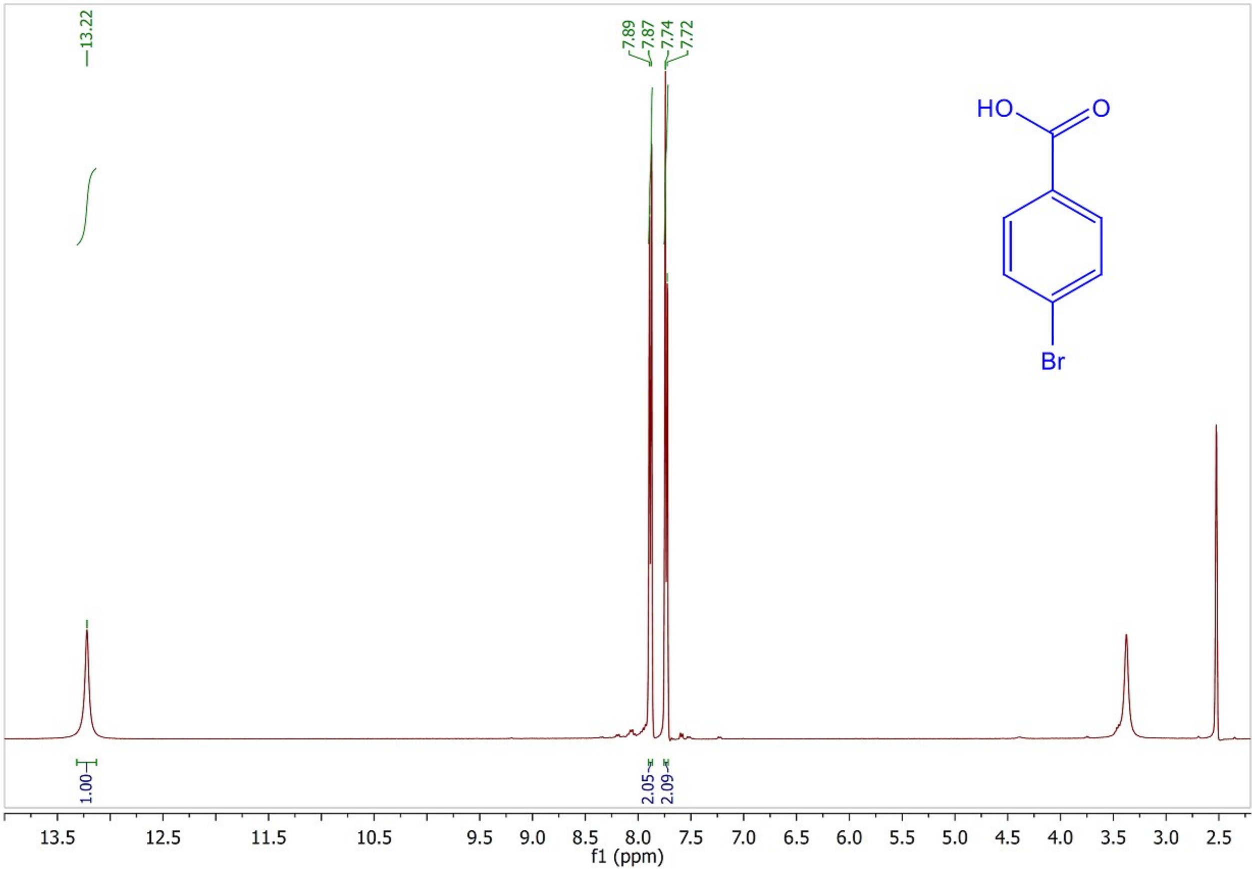

11. 4-Iodobenzoic acid

^{1}H NMR (400 MHz, d_6-DMSO): δ (ppm) = 13.18 (s, 1H), 7.91 (d, *J*= 8 Hz, 2H), 7.71 (d, *J*= 8 Hz, 2H).

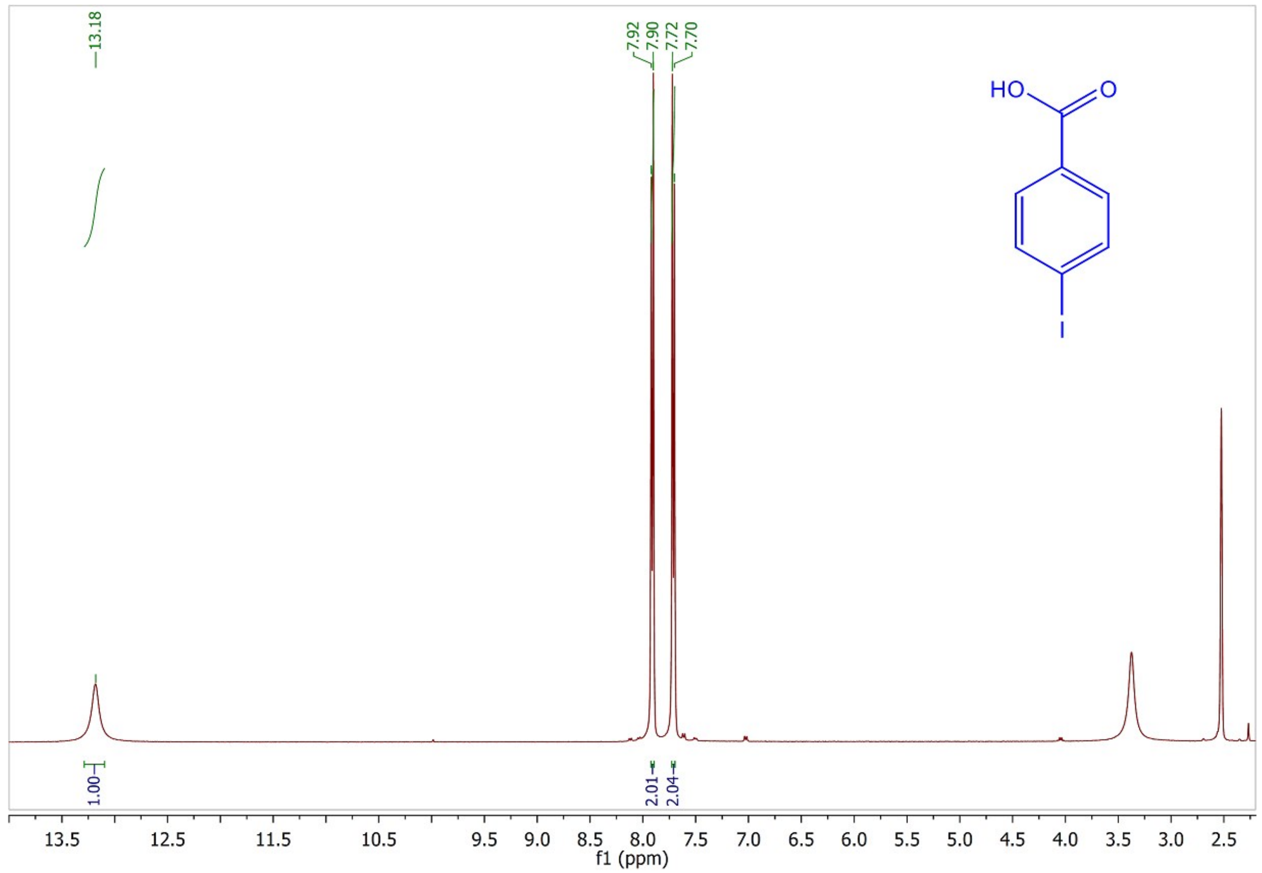

12. 4-Methoxybenzoic acid

^{1}H NMR (400 MHz, d_6-DMSO): δ (ppm) = 12.66 (s, 1H), 7.92 (s, 2H), 7.05 (s, 2H), 3.85 (s, 3H).

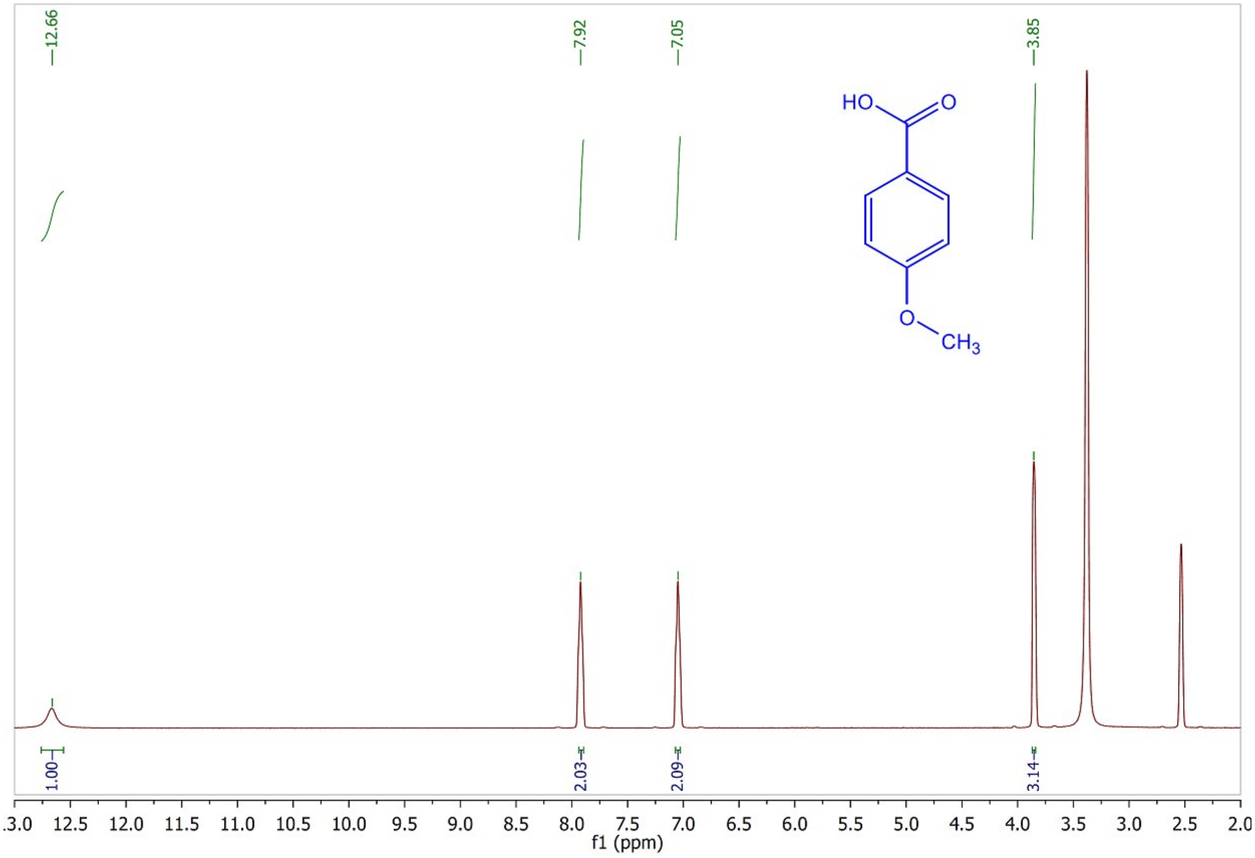

13. 4-Nitrobenzoic acid

^{1}H NMR (400 MHz, d_6-DMSO): δ (ppm) = 13.71 (s, 1H), 8.34 (d, *J*= 8 Hz, 2H), 8.18 (d, *J*= 8 Hz, 2H).

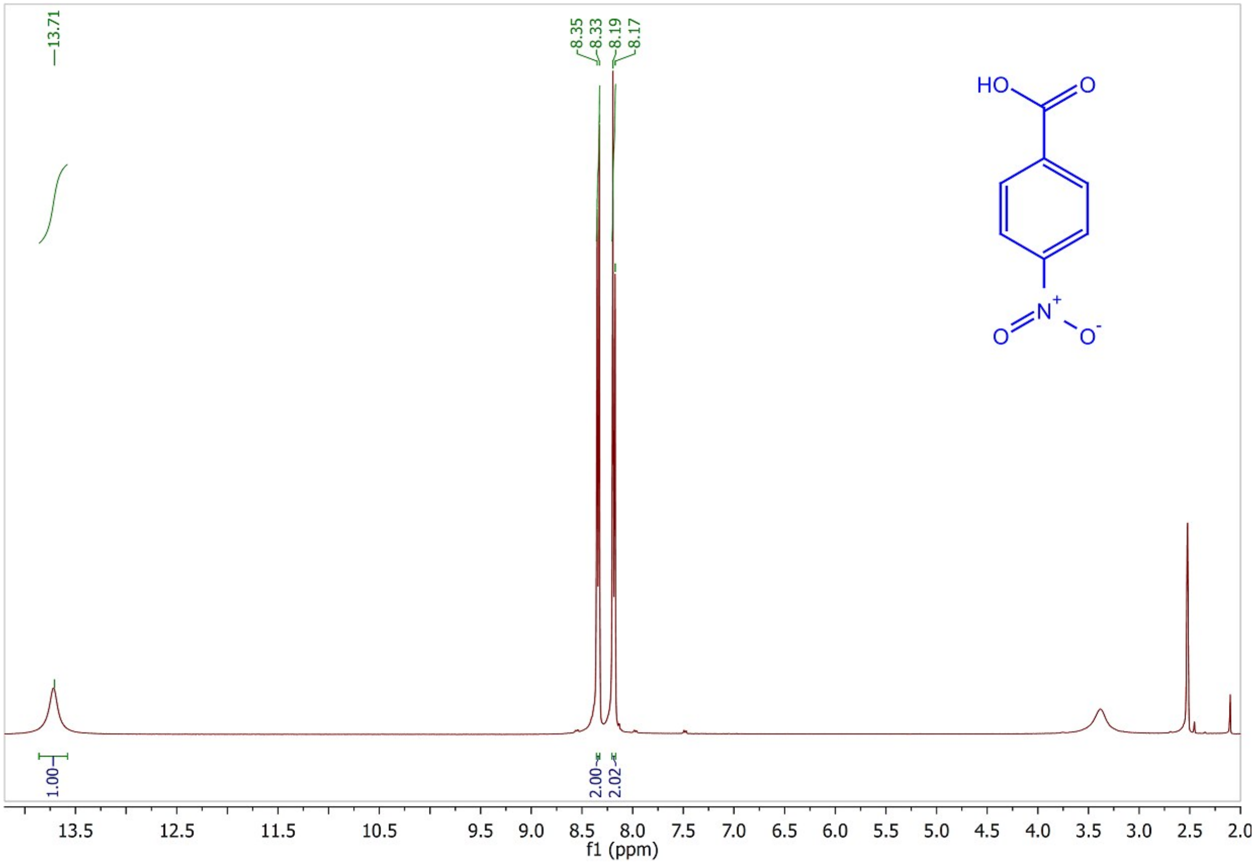

14. Acetophenone

^{1}H NMR (400 MHz, $CDCl_3$): δ (ppm) = 7.89-7.86 (m, 2H), 7.47 (t, *J*= 8 Hz, 1H), 7.36 (t, *J*= 8 Hz, 2H), 2.50 (s, 3H).

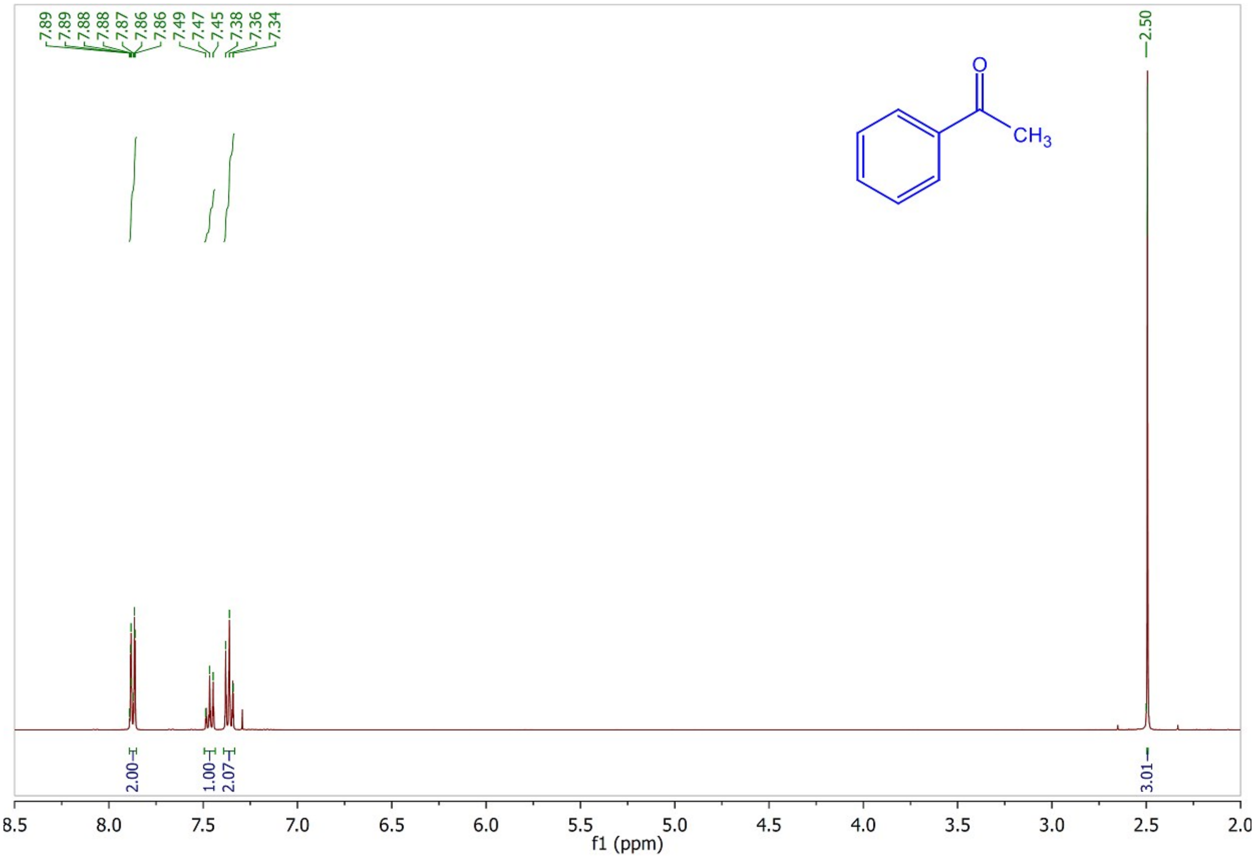

15. Benzophenone

^{1}H NMR (400 MHz, $CDCl_3$): δ (ppm) = 7.85-7.64 (m, 4H), 7.63-7.50 (m, *J*= 6H)).

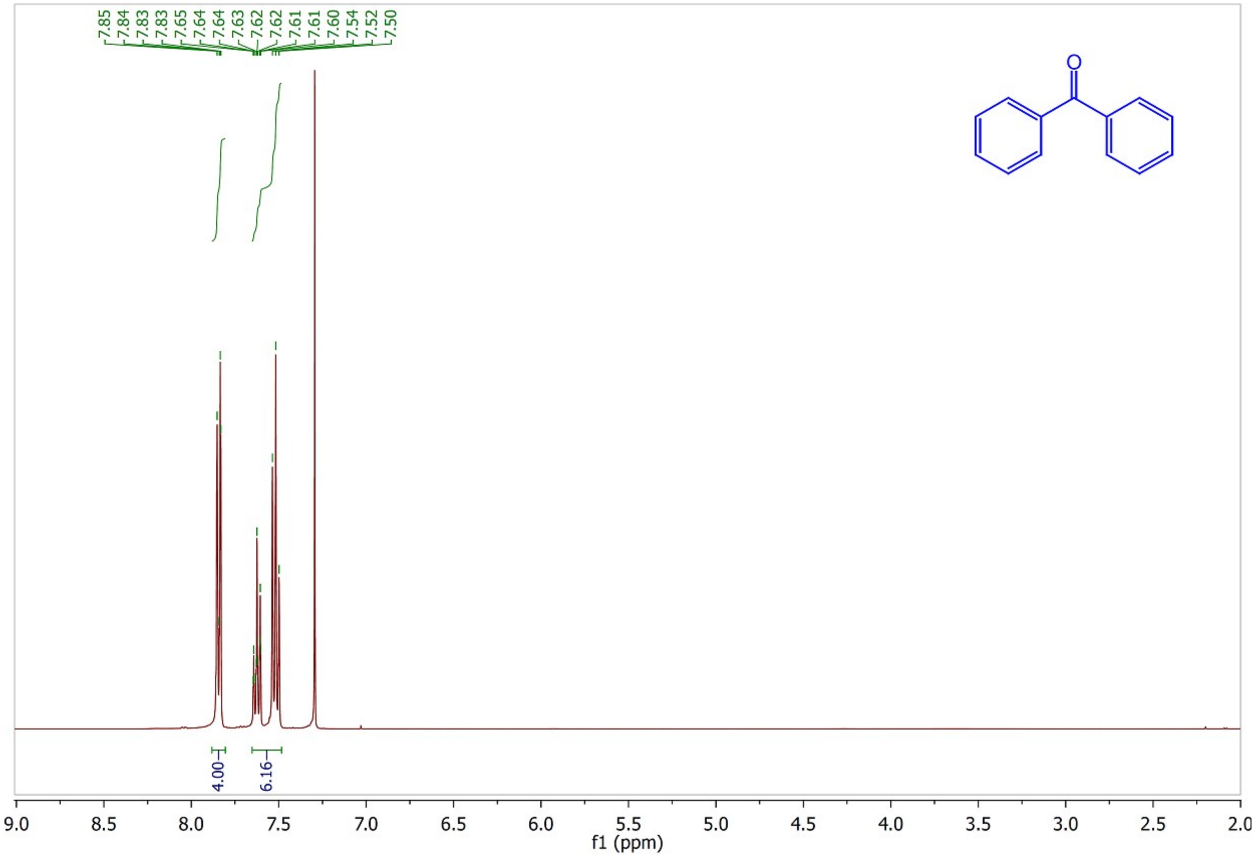

16. Phenyl(pyridin-3-yl)methanone

^{1}H NMR (400 MHz, d_6-DMSO): δ (ppm) = 8.76- 7.31 (m, 9H).

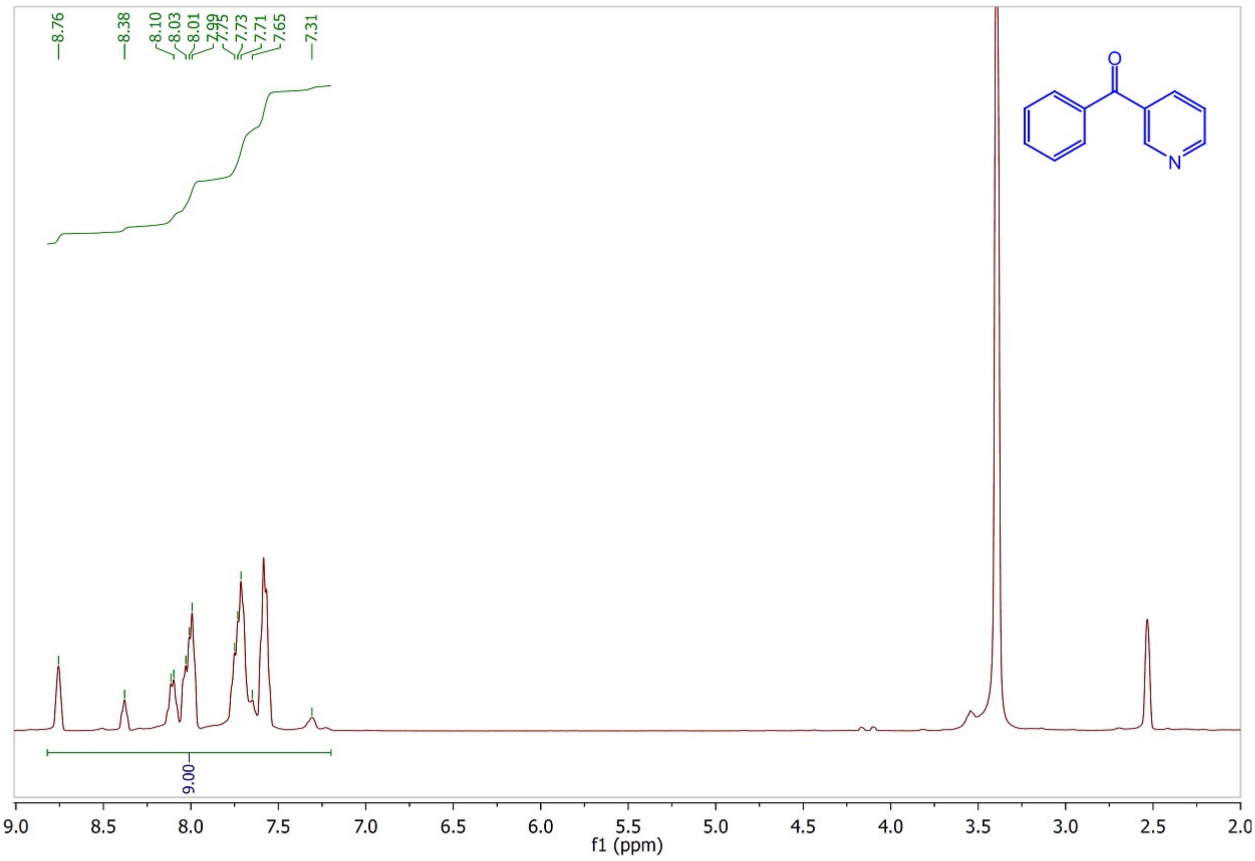

17. 1-Tetralone

^{1}H NMR (400 MHz, $CDCl_3$): δ (ppm) = 8.03 (d, *J*= 8 Hz, 1H), 7.47-7.44 (m, 1H), 7.31- 7.23 (m, 2H), 7.49 (t, *J*= 8 Hz, 1H), 2.97-2.94 (m, 2H), 2.66-2.63 (m, 2H), 2.14-2.11 (m, 2H).

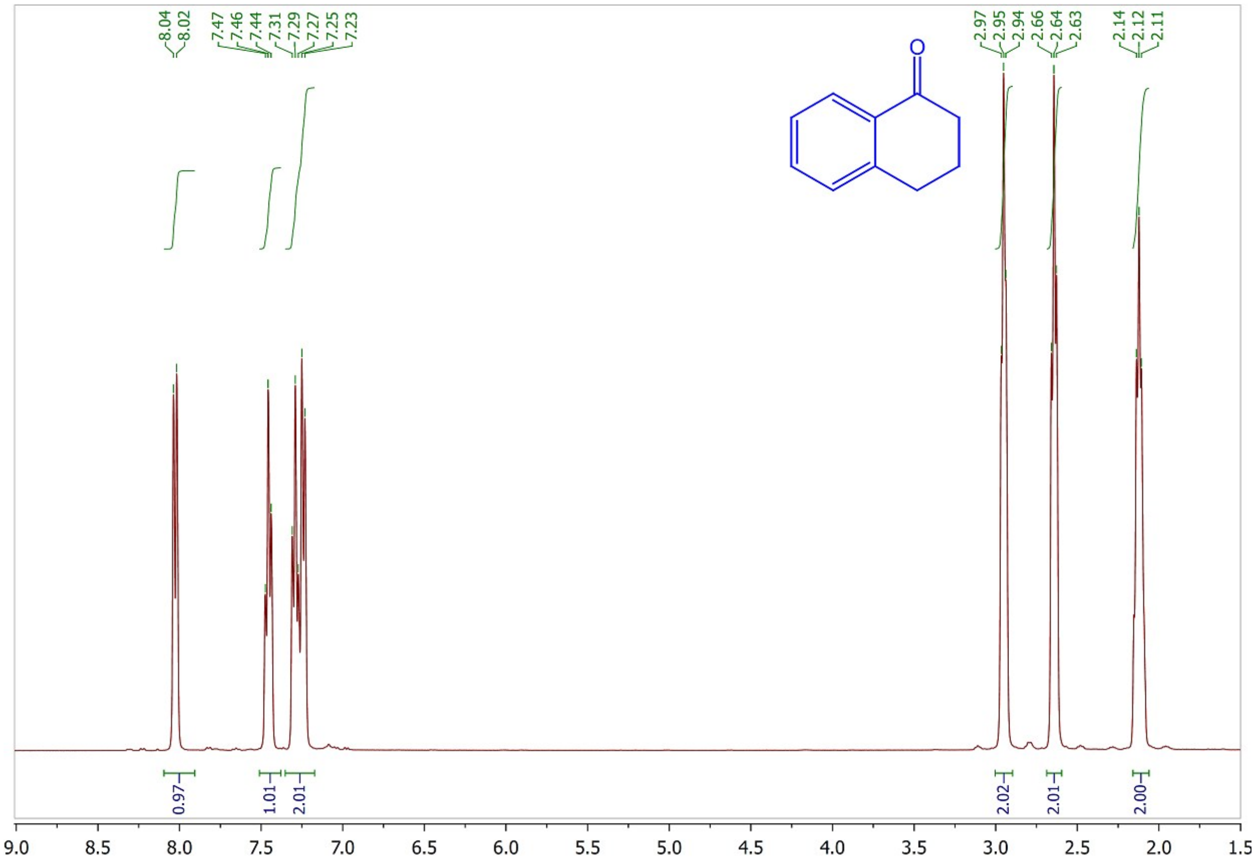

Supplement: RA-010-D0RA03483E-s001 [file RA-010-D0RA03483E-s001.pdf]
